# Genome Annotation Generator: a simple tool for generating and correcting WGS annotation tables for NCBI submission

**DOI:** 10.1093/gigascience/giy018

**Published:** 2018-03-04

**Authors:** Scott M Geib, Brian Hall, Theodore Derego, Forest T Bremer, Kyle Cannoles, Sheina B Sim

**Affiliations:** 1 Tropical Plant Protection Research Unit, USDA-ARS Daniel K. Inouye U.S. Pacific Basin Agricultural Research Center, Hilo, HI, 96720, USA; 2 Plant and Environmental Protection Science, University of Hawaii at Manoa, Honolulu, HI, 96822, USA; 3 Department of Computer Science and Engineering, University of Hawaii at Hilo, Hilo, HI, 96720, USA

**Keywords:** Genome curation, annotation, whole genome sequencing project

## Abstract

**Background:**

One of the most overlooked, yet critical, components of a whole genome sequencing (WGS) project is the submission and curation of the data to a genomic repository, most commonly the National Center for Biotechnology Information (NCBI). While large genome centers or genome groups have developed software tools for post-annotation assembly filtering, annotation, and conversion into the NCBI’s annotation table format, these tools typically require back-end setup and connection to an Structured Query Language (SQL) database and/or some knowledge of programming (Perl, Python) to implement. With WGS becoming commonplace, genome sequencing projects are moving away from the genome centers and into the ecology or biology lab, where fewer resources are present to support the process of genome assembly curation. To fill this gap, we developed software to assess, filter, and transfer annotation and convert a draft genome assembly and annotation set into the NCBI annotation table (.*tbl*) format, facilitating submission to the NCBI Genome Assembly database. This software has no dependencies, is compatible across platforms, and utilizes a simple command to perform a variety of simple and complex post-analysis, pre-NCBI submission WGS project tasks.

**Findings:**

The Genome Annotation Generator is a consistent and user-friendly bioinformatics tool that can be used to generate a *.tbl* file that is consistent with the NCBI submission pipeline

**Conclusions:**

The Genome Annotation Generator achieves the goal of providing a publicly available tool that will facilitate the submission of annotated genome assemblies to the NCBI. It is useful for any individual researcher or research group that wishes to submit a genome assembly of their study system to the NCBI.

## Introduction

While ever-improving sequencing technology and assembly software enable the collection of raw sequences for genome assembly and structural annotation, further steps need to be taken to ensure the quality and completeness of a whole genome sequencing (WGS) project for submission to the National Center for Biotechnology Information (NCBI) or other data repositories [1]. To submit a genome to the NCBI for curation, it must be converted to the NCBI annotation table format (*.tbl*). With a genome assembly project consisting of thousands of sequences demarcated by hundreds of thousands of structural annotations, this task clearly requires automation. However, there is currently no freely available tool that performs rapid and controlled conversion of a genome assembly and associated structural annotations into a *.tbl* format in addition to allowing for editing, modification, and revision of the content of the project. Moreover, the typical assembly and draft annotation contains some degree of questionable or erroneous data that requires correction or omission. It may also be desirable to add functional annotations to the submission and integrate results from InterProScan, Basic Local Alignment Search Tool (BLAST) homology to curated databases, or ontology terms generated by other tools [2–4].

The traditional approach used to address these problems is to use Linux command line tools or write custom scripts that modify and filter the genome using a scripting language such as Perl or Python [5–7] or large-scale genomic database systems [8]. This method may not be easily or readily reproducible or it may be entirely beyond the ability of an investigator who has less familiarity with generating custom scripts *de novo*. Even among those researchers who use best practices to write clean, well-tested, and reusable scripts to accomplish these tasks, doing so requires a large amount of duplicated effort. For this reason, the Genome Annotation Generator (GAG) was written to provide a straightforward and consistent tool for addressing the most common errors in genome assemblies, adding functional annotations from disparate sources, and producing an NCBI submission-ready annotation *.tbl* file. In addition, the software provides a means for integrating existing functional annotations and marking annotations that require manual curation or review. All of these tasks are done through an intuitive command line program that requires only basic Unix skills and has no required dependencies or packages. The program GAG facilitates the submission of WGS projects to NCBI as well as provides a standardized utility and workflow that fosters consistency between projects. Due to emerging genome sequencing initiatives such as the “5,000 Insect Genomes Initiative” (i5k) the Plant Genome Initiative, and Genome 10K (G10K) [9,10], many independent research groups that are not specialized in genome annotation and analysis are generating large genomic datasets and performing genome sequencing projects within their lab. This program can assist in ensuring quality and consistency of data for new genome biologists.

## Overview

The GAG program is a command line Python program, written in Python 2.7 and requiring no additional outside programs or packages to run. The user directs the program to the genome *.fasta* file and a *.gff3* file containing structural annotations. In addition, a number of options can be used to fix possible errors, flag or remove features (i.e., genomic elements described in the *.gff* structural annotation file) based on selected criteria, add functional annotations, trim regions of the genome out of the assembly, and, of course, write the genome to an NCBI *.tbl* file format. In addition, changes made to the genome annotation, functional annotations added, and flags requesting manual review are also annotated back to the *.gff3* structural annotation file, and the original .*fasta* file is corrected as needed. When the user issues commands to modify the genome, e.g., to remove short introns, the statistics will display 2 columns, representing the original and modified genomes. This allows for stepwise and documented filtering and review to occur and for interactions between GAG and visual genome review tools (e.g., Artemis, Apollo, GBrowse, JBrowse, etc.) [11–15].

## Methods

As an example, we consider a possible workflow for a user wishing to prepare a genome for submission to the NCBI Eukaryotic WGS Database. The user has a scaffolded genome assembly produced by one of many whole genome assemblers [16–18] in *.fasta* file format and a corresponding GFF3 feature file [19, 20] containing structural annotations resulting from an automated annotation pipeline or predictors such as Maker, Evidence Modeler, Jigsaw, and others see [21–28]. The approach would be to first possibly generate functional annotations of predicted genes if this is desired, using whatever approach the user is interested in, and then using the genome and annotations with GAG. After using GAG to remove or flag features of interest, the user may then further investigate flagged features in a genome browser by loading the output of GAG, editing, and then performing further filtering in GAG, and iterate through this process until a final draft genome product is generated. Finally GAG writes an NCBI table file, on which *tbl2asn* is run for submission to the NCBI. This may identify regions of the genome that need to be trimmed, due to possible adapter contamination in the genome or low-quality sequence. Any errors generated by *tbl2asn* can then be corrected in GAG and the genome trimmed, until an error-free submission is generated.

To use GAG, the user creates a folder containing the genome files (or links to them) and runs *gag.py* from the terminal with the *.fasta* and *.gff3* files. GAG will write a statistics file containing information on the number of each feature type, lengths, and other information that may be useful for the submitter. In our experience, automated genome annotation software frequently produces assemblies containing introns as short as 1 base pair (bp) long; if any such features are present, GAG can be run to detect them. It is important to note than while the NCBI requires short introns to be removed, cutoffs recommended by NCBI may be more stringent than what you want, as they are set to reduce the amount of erroneous data being entered into NCBI. For example, prediction of single base introns might not be errors and may represent true data. It is up to the user to dictate what cutoffs they want to define to remove or flag for manual review. To address these short introns, the user simply applies option *-ris* (REMOVE_INTRONS_SHORTER_THAN) with a value of 10 bp. GAG will discard any mRNA containing an intron shorter than the minimum of 10 bp. A comparison of the genome content before and after removal is printed to the *.stats* file. If the user instead wishes to only flag features that meet these criteria and not remove them, alternatively the *-fis* (FLAG_INTRONS_SHORTER_THAN) option could be used, which instead adds a *GAG_FLAG* annotation to the attributes column of the *.gff3* file describing the reason for flagging, allowing manual review of flagged features in a genome browser. GAG will automatically update all parent and child features (gene or coding sequence (CDS) entries) to reflect removal of mRNA features. Available flag or removal options are listed in Table 1.

**Table 1:** Options for GAG

Option	Type of function	Description
*-a* <annotation file>	Annotate	Adds functional annotations present in annotation file to *.gff* and *.tbl*
*-t* <*.bed* file>	Trim	Removes regions of genome indicated in *.bed* file from *.fasta* and *.gff3*
*-fix_start_stop* <no value>	Fix	Adds or corrects start and stop codon features to *.gff3*
*-fix_terminal_ns* <no value>	Fix	Removes any trailing ends from contig ends in assembly, updates *.gff3* coordinates
*-rcs* <integer>	Remove	Remove CDS shorter than <integer>
*-rcl* <integer>	Remove	Remove CDS longer than <integer>
*-res* <integer>	Remove	Remove exons shorter than <integer>
*-rel* <integer>	Remove	Remove exons longer than <integer>
*-ris* <integer>	Remove	Remove introns shorter than <integer>
*-ril* <integer>	Remove	Remove introns longer than <integer>
*-rgs* <integer>	Remove	Remove genes shorter than <integer>
*-rgl* <integer>	Remove	Remove genes longer than <integer>
*-fcs* <integer>	Flag	Flag CDS shorter than <integer>
*-fcl* <integer>	Flag	Flag CDS longer than <integer>
*-fes* <integer>	Flag	Flag exons shorter than <integer>
*-fel* <integer>	Flag	Flag exons longer than <integer>
*-fis* <integer>	Flag	Flag introns shorter than <integer>
*-fil* <integer>	Flag	Flag introns longer than <integer>
*-fgs* <integer>	Flag	Flag genes shorter than <integer>
*-fgl* <integer>	Flag	Flag genes longer than <integer>

Another review for submission might be that all coding regions be a minimum length. For this example we use 150 bp in length, which is suggested by NCBI [29,30]. To add this additional level of filtering, a second option can be used: *-rcs 150*, to REMOVE_CDS_SHORTER_THAN 150 bp. When the genome is written to the output folder, GAG will write a file called *genome.removed.gff* containing all the features left out of the final version. It is important to remember that CDS cutoffs at 150 bp will possibly remove some biologically correct amino acids.

GAG supports 2 straightforward correction, or fix, tools. If the user’s GFF3 file does not explicitly indicate the presence of start and stop codons or if there is reason to believe there are errors in ORF prediction in the provided GFF file, GAG can a add start and stop feature to the GFF file. The user simply issues the command with the option *–fix_start_stop* and these features will be added to the GFF3 file and their existence noted in the table file. A second issue that can arise in a draft genome assembly is for a contig or scaffold to have a string of ambiguous bases (Ns) at the very beginning or end of the contig. These should be removed from the assembly; this can be done using the *–fix_terminal_ns* option, as they can be misinterpreted as scaffold gaps. Removing these regions from the genome will disrupt the parity between coordinates in the *.fasta* genome file and the *.gff3* annotation file. GAG will automatically update coordinates in the *.gff3* file to reflect any regions removed from the sequence file. During execution of *tbl2asn* or submission to NCBI, it may be identified that regions of the genome may be contaminated with a microbial, vector, or sequencing adapter sequence as part of the “contaminate screen” step. A *.bed* formatted file can be supplied with the *-trim* option, containing regions of the assembly to exclude, either ranges within a contig or scaffold, or an entire scaffold. GAG will update both the *.fasta* and *.gff3* files so that coordinates are still synchronized. This is a particularly difficult operation to perform without a specialized tool.

At present, GAG has simple commands to remove or flag introns, exons, coding regions, and genes based on minimum or maximum lengths, which will also edit or remove any parent or child feature from the annotation file so as not to create incomplete feature annotations. It can also remove features from a list, which is useful in cases where a genome submission is rejected and a list of invalid mRNAs and genes is provided. In addition, all discarded features are retained in a “genome.removed.gff” file, and the entire editing session is documented so that the user can retain the filtering criteria used on the particular dataset.

GAG supports 2 methods to add functional annotations to a genome. First, it can read an annotated GFF3 file containing gene names, protein products, cross-references to databases, and ontology terms following GFF3 qualified nomenclature in the *attribute* column of the GFF3 file [31–34]. Any annotations present will be automatically carried over to the NCBI feature table file. For users with annotations from another source, GAG can read them from a simple tab-delimited file. The annotations supported by the current version of GAG are *Name* (for genes), *Dbxref*, *Ontology_term*, and *product* (for descriptive mRNA products). These are also written to a new GFF3 file, so GAG can be utilized as a tool to also functionally annotate a GFF3 file. Detailed instructions for running GAG, examples for each of the main functions (e.g., removing features, adding start and stop codons, trimming features, adding annotations) as well as formats and conversion tools for functional annotations are available on the GAG software website webpage: http://genomeannotation.github.io/GAG/, and a stable release (version 2.0.1) is available in an accompanying *Gigascience* database and Code Ocean entries [35,36] and is shared through SciCrunch under (Genome Annotation Generator, RRID:SCR_016053).

## Implementation

GAG is written in Python 2.7. It has no dependencies beyond the standard library. The program is modular, abstracting biological concepts such as Sequence, Gene, and CDS into classes that may be incorporated into other software tools. In addition, the code is covered by a suite of unit and integration tests, allowing developers to modify or add to the code base with reduced risk of introducing errors. It should be easily executable by the novice programmer with only basic command-line experience but also powerful enough to be implemented within robust genomic data processing pipelines.

## Conclusion

GAG can be easily expanded in the future to support more specific needs of researchers and less common annotation types and to integrate conversion of common functional annotation output formats (e.g., InterProScan, BLAST, Blast2Go) for addition to NCBI annotation table formats. Currently, GAG is an intermediate but critical tool, it fits between a simple format conversion tool and more sophisticated annotation editors. In future developments of GAG, we plan to allow the integration of multiple lines of evidence supporting gene models to help users discriminate apparently high-quality annotations from annotations with little support or possible errors. This could rapidly improve and standardize manual annotation efforts in systems and user groups that are not integrated into genome center annotation pipelines.

### Availability of supporting data

Snapshots of the software and accompanying files are available from the GigaScience GigaDB database [35], and the algorithm is also accessible in the Code Ocean cloud-based computational reproducibility platform [36] https://codeocean.com/2018/02/06/genome-annotation-generator-ncbi-for-submission/.

### Availability and requirements

- Project name: Genome Annotation Generator
- Project home page: https://github.com/genomeannotation/GAG
- Operating systems: Linux, Windows, OS
- Programming language: Python
- Other requirements: Python 2
- License: MIT
- SciCrunch RRID:SCR_016053

## Abbreviations

BLAST: Basic Local Alignment Search Tool; bp: base pair; CDS: coding sequence; GAG: Genome Annotation Generator; NCBI: National Center for Biotechnology Information; WGS: whole genome sequencing

## Competing Interests

The authors declare that they have no competing interests.

## Funding

Funding for this project was provided by US Department of Agriculture (USDA)-Agricultural Research Service (ARS) and USDA-Animal and Plant Health Inspection Service (APHIS) Farm Bill Section 10007 projects 3.0251.02 (FY 2014), 3.0256.01 (FY 2015), 3.0392.02 (FY 2016).

## Author contributions

S.M.G. conceived software concept. B.H., T.D., and S.M.G. designed and wrote the software. B.H., S.M.G., and S.B.S. wrote the manuscript.

## Supplementary Material

GIGA-D-17-00030_Original_Submission.pdf

GIGA-D-17-00030_Revision_1.pdf

GIGA-D-17-00030_Revision_2.pdf

Response_to_Reviewer_Comments_Original_Submission.pdf

Response_to_Reviewer_Comments_Revision_1.pdf

Reviewer_1_Report_(Original_Submission) -- Kristoffer Forslund 28 Feb 2017 Reviewed

Reviewer_2_Report_(Original_Submission) -- Juan Oliveros 08 Mar 2017 Reviewed

Reviewer_3_Report_(Original_Submission) -- Monica Munoz-Torres 15 Mar 2017 Reviewed
