# Supplementary material for: Genome Annotation Generator: a simple tool for generating and correcting WGS annotation tables for NCBI submission
Source: Gigascience. 2018 Mar 4;7(4):giy018. doi: 10.1093/gigascience/giy018 (PMC5887294; doi:10.1093/gigascience/giy018)
Supplement: GIGA-D-17-00030_Original_Submission.pdf [file giy018_giga-d-17-00030_original_submission.pdf]

GigaScience

Genome Annotation Generator: A simple tool for generating and correcting WGS annotation tables for NCBI submission

--Manuscript Draft--

**Manuscript Number:**	GIGA-D-17-00030
**Full Title:**	Genome Annotation Generator: A simple tool for generating and correcting WGS annotation tables for NCBI submission
**Article Type:**	Technical Note
**Funding Information:**	Agricultural Research Service — Dr Scott M Geib
**Abstract:**	One of the most overlooked, yet critical components of a whole genome sequencing project is the submission and curation of the data to a genomic repository, most commonly NCBI. While large genome centers or genome groups have developed software tools for post-annotation assembly filtering, annotation, and conversion into NCBI's annotation table format, these tools typically require back-end setup and connection to an SQL database and/or some knowledge of programming (Perl, Python) to implement. With whole genome sequencing becoming commonplace, genome sequencing projects are moving away from the genome centers, and into the ecology or biology lab, where much less resources are present to support the process of genome assembly curation. To fill this gap, we developed software to assess, filter, transfer annotations, and convert a draft genome assembly and annotation set into NCBI annotation table (.tbl) format, facilitating submission to NCBI Genome Assembly database. This software has no dependencies, is compatible across platforms, and utilizes a simple command line to perform a variety of simple and complex post-analysis, pre-NCBI submission WGS project tasks.
**Corresponding Author:**	Scott M Geib<br><br>UNITED STATES
**Corresponding Author Secondary Information:**	
**Corresponding Author's Institution:**	
**Corresponding Author's Secondary Institution:**	
**First Author:**	Scott M Geib
**First Author Secondary Information:**	
**Order of Authors:**	Scott M Geib
	Brian Hall
	Theodore DeRego
	Sheina B Sim
**Order of Authors Secondary Information:**	
**Opposed Reviewers:**	
**Additional Information:**	
**Question**	**Response**
Are you submitting this manuscript to a special series or article collection?	No
**Experimental design and statistics**<br><br>Full details of the experimental design and	Yes

Powered by Editorial Manager® and ProduXion Manager® from Aries Systems Corporation

statistical methods used should be given in the Methods section, as detailed in our Minimum Standards Reporting Checklist. Information essential to interpreting the data presented should be made available in the figure legends.<br><br>Have you included all the information requested in your manuscript?	
**Resources**<br><br>A description of all resources used, including antibodies, cell lines, animals and software tools, with enough information to allow them to be uniquely identified, should be included in the Methods section. Authors are strongly encouraged to cite Research Resource Identifiers (RRIDs) for antibodies, model organisms and tools, where possible.<br><br>Have you included the information requested as detailed in our Minimum Standards Reporting Checklist?	Yes
**Availability of data and materials**<br><br>All datasets and code on which the conclusions of the paper rely must be either included in your submission or deposited in publicly available repositories (where available and ethically appropriate), referencing such data using a unique identifier in the references and in the "Availability of Data and Materials" section of your manuscript.<br><br>Have you have met the above requirement as detailed in our Minimum Standards Reporting Checklist?	Yes

Manuscript

Click here to download Manuscript genome-annotation-generator.pdf

Genome Annotation Generator: A simple tool for generating and correcting WGS annotation tables for NCBI submission

Scott M. Geib [1*†], Brian Hall [2†], Theodore Derego [1], and Sheina B. Sim [1,2]

[1]Tropical Plant Protection Research Unit, USDA-ARS Daniel K. Inouye US Pacific Basin Agricultural Research Center, Hilo, HI, 96720, USA

[2]Plant and Environmental Protection Science, University of Hawaii at Manoa, Honolulu, HI, 96822, USA.

*Corresponding author †Authors contributed equally

email: SMG: scott.geib@ars.usda.gov BH:bhall7@hawaii.edu TD:t.derego@yahoo.com SBS:sheina.sim@ars.usda.gov

February 8, 2017

Abstract

Background

One of the most overlooked, yet critical components of a whole genome sequencing project is the submission and curation of the data to a genomic repository, most commonly NCBI. While large genome centers or genome groups have developed software tools for post-annotation assembly filtering, annotation, and conversion into NCBI's annotation table format, these tools typically require back-end setup and connection to an SQL database and/or some knowledge of programming (Perl, Python) to implement. With whole genome sequencing becoming commonplace, genome sequencing projects are moving away from the genome centers, and into the ecology or biology lab, where much less resources are present to support the process of genome assembly curation. To fill this gap, we developed software to assess, filter, transfer annotations, and convert a draft genome assembly and annotation set into NCBI annotation table (.tbl) format, facilitating submission to NCBI Genome Assembly database. This software has no dependencies, is compatible across platforms, and utilizes a simple command line to perform a variety of simple and complex post-analysis, pre-NCBI submission WGS project tasks.

Findings

The Genome Annotation Generator is a consistent and user-friendly bioinformatics tool that can be used to generate a *.tbl* file that is consistent with the NCBI submission pipeline.

Conclusions

The Genome Annotation Generator achieves the goal of providing a publicly available tool that will facilitate the submission of annotated genome assemblies to NCBI.

It is useful for any individual researcher or research group who wishes to submit a genome assembly of their study system to NCBI.

Keywords: Genome curation; annotation; and whole-genome sequencing project

1 Introduction

While ever-improving sequencing technology and assembly software enable the collection of raw sequences for genome assembly and structural annotation, further steps need to be taken to ensure the quality and completeness of a WGS project for submission to the National Center for Biotechnology Information (NCBI) or other data repositories [32]. To submit a genome to the NCBI for curation, it must be converted to the NCBI annotation table format (*.tbl*). With a genome assembly project consisting of thousands of sequences demarcated by hundreds of thousands of structural annotations, this task clearly requires automation. However, there is currently no freely available tool which performs rapid and controlled conversion of a genome assembly and associated structural annotations into a *.tbl* format in addition to allowing for editing, modification, and revision of the content of the project. Moreover, the typical assembly and draft annotation contains some degree of questionable or erroneous data which requires correction or omission. It may also be desirable to add functional annotations to the submission and integrate results from InterProScan, BLAST homology to curated databases, or ontology terms generated by other tools [33, 5, 17].

The traditional approach used to address these problems is to use Linux command line tools or write custom scripts which modify and filter the genome using a scripting language such as Perl or Python [4, 28, 12] or large scale genomic database systems [20]. This method may not be easily or readily reproducible, or it may be entirely beyond the ability of an investigator who has less familiarity with generating custom scripts *de novo*. Even amongst those researchers who use best practices to write clean, well-tested, and reusable scripts to accomplish these tasks, doing requires a large amount of duplicated effort. For this reason, the Genome Annotation Generator (GAG) was written to provide a straightforward and consistent tool for addressing the most common errors in genome assemblies, adding functional annotations from disparate sources, and producing an NCBI submission-ready annotation *.tbl* file. In addition, the software provides a means for integrating existing functional annotations and marking annotations that require manual curation or review. All of these tasks are done through an intuitive command line program, a friendly user-interface, and has no required dependencies or packages. The program GAG facilitates the submission of whole genome sequencing (WGS) projects to NCBI as well as provide a standardized utility and workflow that fosters consistency between projects. Due to emerging genome sequencing initiatives such as the 5,000 Insect

Genomes Initiative (i5K), the Plant Genome Initiative, and Genome 10K [22, 21], many independent research groups which are not specialized in genome annotation and analysis are generating large genomic datasets and performing genome sequencing projects within their lab. This program can assist in ensuring quality and consistency of data for new genome biologists.

2 Overview

The GAG program is a command line python program, written in python 2.7 and requiring no additional outside programs or packages to run. The user directs the program to the genome *.fasta* file and a *.gff3* file containing structure annotations. In addition, a number of flags can be used to fix possible errors, flag or remove features based on selected criteria, add functional annotations, trim regions of the genome out of the assembly, and, of course, write the genome to NCBI *.tbl* file format. In addition, changes made to the genome annotation, functional annotations added, or flags requesting manual review are also annotated back to the GFF3 structural annotation file, and the original fasta file is corrected as needed. When the user issues commands to modify the genome, e.g. to remove short introns, the statistics will display two columns, representing the original and modified genomes. This allows for stepwise and documented filtering and review to occur, and interactions between GAG and visual genome review tools (e.g. Artemis, Apollo, Gbrowse) [31, 25, 16, 27, 24].

3 Methods

As an example, we consider a possible work-flow for a user wishing to prepare a genome for submission to the NCBI Eukaryotic WGS Database. She has a scaffolded genome assembly produced by one of many whole genome assemblers [2, 13, 26] in *.fasta* file format and a corresponding GFF3 feature file [8, 7] containing structural annotations resulting from an automated annotation pipeline or predictors such as Maker, Evidence Modeler, Jigsaw, or others e.g. [3, 15, 1, 14, 29, 30, 6, 9]. The approach would be to first possibly generate functional annotations of predicted genes if this is desired, using whatever approach the user is interested in, and then using the genome and annotations with GAG. After using GAG to remove or flag features of interest, she then may then further investigate flagged features in a genome browser by loading the output of GAG, edit, and then perform further filtering in GAG, and iterate through this process until a final draft genome product is generated. Finally GAG writes a NCBI table file, on which tbl2asn is run for submission to NCBI. This may identify regions of the genome that need to be trimmed, due to possible adapter contamination in the genome, or low quality sequence. Any errors generated by tbl2asn can then be corrected in GAG, the

genome trimmed, until an error free submission is generated.

To use GAG, she creates a folder containing the genome files (or links to them) and runs *gag.py* from the terminal, with the *.fasta* and *.gff3* files. GAG will write a statistics file, containing infomraiton on the number of each feature type, lengths, and other information that may be useful for the submitter. In our experience, automated genome annotation software frequently produces assemblies containing introns as short as 1 base pair long; if any such features are present, GAG will detect them. It is common for NCBI to request that genes containing short introns be removed or modified, and GAG can be used to do this. To address these short introns, the user simply applies flag *-ris* (Remove_Intron_Shorter_Than) with a value of *10*. GAG will discard any mRNA containing an intron shorter than the minimum of ten. A comparison of the genome content before and after removal is printed to the *.stats* file. If she instead wishes to only flag features that meet this criteria and not remove them, alternatively the *-fis* (Flag_Introns_Shorter_Than) flag could be used, which instead adds a *GAG_FLAG* feature to the attributes column of the *.gff3* file describing the reason for flagging, allowing manual review of flagged features in a genome browser. GAG will automatically update all parent and child features (gene or CDS entries) to reflect removal of mRNA features. A list of available flag or removal options are listed in Table 1.

Another review for submission might be that all coding regions be a minimum length. For this example we use 150 base pairs in length, which is suggested by NCBI [10, 11]. The to add this additional level of filtering, a second flag can be used: *-rcs 150*, to Remove_CDS_Shorter_Than 150 bp. When the genome is written to the output folder, GAG will write a file called *genome.removed.gff* containing all the features left out of the final version.)

GAG supports two straightforward correction, or fix tools. If the user's GFF3 file does not explicitly indicate the presence of start and stop codons, or if there is reason to believe there are errors in ORF prediction, GAG can calculate start and stop locations. The user simply issues the command with the flag *–fix_start_stop* and these features will be added to the GFF3 file, and their existence noted in the table file. A second issue that can arise in a draft genome assembly is for a contig or scaffold to have a string of ambiguous bases (N's) at the very beginning or end of the contig. These should be removed from the assembly, and can be using the *–fix_terminal_ns* flag, as they can be mis-interpreted as scaffold gaps. Removing these regions from the genome though, will disrupt the parity between coordinates in the *.fasta* genome file and the *.gff3* annotation file. GAG will automatically update coordinates in the *.gff3* file to reflect any regions removed from the sequence file. In addition, it may be identified that regions of the genome may be contaminated with microbial, vector, or sequencing adapter sequence, particularly identified during the "contaminate screen" performed during execution of *tbl2asn* and NCBI quality checking. A *.bed* formatted file can be supplied with teh -

trim flag, containing regions of the assembly to exclude, either ranges within a contig or scaffold, or an entire scaffold. GAG will update both the *.fasta* and *.gff3* files so that coordinate are still synchronized. This is a particularly difficult operation to perform without a specialized tool.

At present, GAG has simple commands to remove or flag introns, exons, coding regions and genes based on minimum or maximum lengths, which will also edit or remove any parent or child feature from the annotation file so as not to create incomplete feature annotations. It can also remove features from a list, which is useful for cases where a genome submission is rejected and a list of invalid mRNAs and genes provided. In addition, all discarded features are retained in a "genome.removed.gff" file and the entire editing session is documented so that the user can retain the filtering criteria used on the particular dataset.

GAG supports two methods to add functional annotations to a genome. First, it can read an annotated GFF3 file containing gene names, protein products, cross-references to databases, and ontology terms following GFF3 qualified nomenclature in the *attribute* column of the GFF3 file [19, 18, 23]. Any annotations present will be automatically carried over to the NCBI feature table file. For users with annotations from another source, GAG can read them from a simple tab-delimited file. The annotations supported by the current version of GAG are *Name* (for genes), *Dbxref*, *Ontology_ term* and *product* (for descriptive mRNA products). These are also written to a new GFF3 file, so GAG can be utilized as a tool to also functionally annotate a GFF3 file. Detailed instructions for running GAG, examples, as well as formats and conversion tools for functional annotations are available on the GAG software website webpage: `http://genomeannotation.github.io/GAG/`.

4 Implementation

GAG is written in Python 2.7. It has no dependencies beyond the standard library. The program is modular, abstracting biological concepts such as Sequence, Gene and CDS into classes which may be incorporated into other software tools. In addition, the code is covered by a suite of unit and integration tests, allowing developers to modify or add to the code base with reduced risk of introducing errors. It should be easily executable by the novice programmer, but also powerful enough to be implemented within robust genome processing pipelines.

5 Conclusion

GAG can be easily expanded in the future to support more specific needs of researchers, less common annotation types, and integrate conversion of common functional annotation output formats (e.g. InterProScan, BLAST, Blast2Go) for addition to NCBI annotation

table formats. Currently, GAG is an intermediate, but critical tool, between a simple format conversion tool and more sophisticated annotation editors. In future developments of GAG, we plan to allow the integration of multiple lines of evidence supporting gene models to help users discriminate apparently high quality annotations from annotations with little support or possible errors. This could rapidly improve and standardize manual annotation efforts in systems and user groups that are not integrated into genome center annotation pipelines.

6 Declarations

6.1 Competing Interests

The authors declare that they have no competing interests

6.2 Funding

Funding for this project was provided by USDA-ARS and USDA-APHIS Farm Bill Section 10007 projects 3.0251.02 (FY 2014), 3.0256.01 (FY 2015), 3.0392.02 (FY 2016).

6.3 Authors' contributions

SMG conceived software concept. BH, TD, and SMG designed and wrote software. BH, SMG, and SBS wrote manuscript.

6.4 Acknowledgements

We thank S. Gayle, B. Calla, and others for assisting in beta testing of the software and making test datasets available to us. Bioinformatic analysis to develop test datasets for GAG was performed on computing resources at USDA-ARS Pacific Basin Agricultural Research Center (Moana cluster; Hilo, HI) and the Extreme Science and Engineering Discovery Environment (XSEDE), which is supported by National Science Foundation grant number OCI-1053575XSEDE utilizing allocation TG-MCB140032 to S.M.G. Opinions, findings, conclusions, or recommendations expressed in this publication are those of the authors and do not necessarily reflect the views of the USDA. USDA is an equal opportunity provider and employer

References

[1] Jonathan E. Allen and Steven L. Salzberg. Jigsaw: integration of multiple sources of evidence for gene prediction. *Bioinformatics*, 21(18):3596–3603, 2005.

Table 1: **Options for GAG**

Option	Type of function	Description
-*a* <annotation file>	Annotate	Adds functional annotations present in annotation file to *.gff* and *.tbl*
-*t* <*.bed* file>	Trim	Removes regions of genome indicated in *.bed* file from *.fasta* and *.gff3*
-*fix_start_stop* <no value>	Fix	Adds or corrects start and stop codon features to *.gff3*
-*fix_terminal_ns* <no value>	Fix	Removes any trailing ends from contig ends in assembly, updates *.gff3* coordinates
-*rcs* <integer>	Remove	Remove CDS shorter than <integer>
-*rcl* <integer>	Remove	Remove CDS longer than <integer>
-*res* <integer>	Remove	Remove exons shorter than <integer>
-*rel* <integer>	Remove	Remove exons longer than <integer>
-*ris* <integer>	Remove	Remove introns shorter than <integer>
-*ril* <integer>	Remove	Remove introns longer than <integer>
-*rgs* <integer>	Remove	Remove genes shorter than <integer>
-*rgl* <integer>	Remove	Remove genes longer than <integer>
-*fcs* <integer>	Flag	Remove CDS shorter than <integer>
-*fcl* <integer>	Flag	Remove CDS longer than <integer>
-*fes* <integer>	Flag	Remove exons shorter than <integer>
-*fel* <integer>	Flag	Remove exons longer than <integer>
-*fis* <integer>	Flag	Remove introns shorter than <integer>
-*fil* <integer>	Flag	Remove introns longer than <integer>
-*fgs* <integer>	Flag	Remove genes shorter than <integer>
-*fgl* <integer>	Flag	Remove genes longer than <integer>

[2] J. Butler, I. MacCallum, M. Kleber, I. A. Shlyakhter, M. K. Belmonte, E. S. Lander, C. Nusbaum, and D. B. Jaffe. Allpaths: de novo assembly of whole-genome shotgun microreads. *Genome Res*, 18(5):810–20, 2008.

[3] Brandi L. Cantarel, Ian Korf, Sofia M. C. Robb, Genis Parra, Eric Ross, Barry Moore, Carson Holt, Alejandro Sanchez Alvarado, and Mark Yandell. Maker: An easy-to-use annotation pipeline designed for emerging model organism genomes. *Genome Research*, 18(1):188–196, 2008. Times Cited: 80.

[4] Peter J. A. Cock, Tiago Antao, Jeffrey T. Chang, Brad A. Chapman, Cymon J. Cox, Andrew Dalke, Iddo Friedberg, Thomas Hamelryck, Frank Kauff, Bartek Wilczynski, and Michiel J. L. de Hoon. Biopython: freely available python tools for computational molecular biology and bioinformatics. *Bioinformatics*, 25(11):1422–1423, 2009.

[5] Ana Conesa, Stefan Götz, Juan Miguel García-Gómez, Javier Terol, Manuel Talón, and Montserrat Robles. Blast2go: a universal tool for annotation, visualization and analysis in functional genomics research. *Bioinformatics*, 21(18):3674–3676, 2005.

[6] Val Curwen, Eduardo Eyras, T. Daniel Andrews, Laura Clarke, Emmanuel Mongin, Steven M.J. Searle, and Michele Clamp. The ensembl automatic gene annotation system. *Genome Research*, 14(5):942–950, 2004.

[7] K. Eilbeck and S. E. Lewis. Sequence ontology annotation guide. *Comp Funct Genomics*, 5(8):642–7, 2004.

[8] Karen Eilbeck, Suzanna Lewis, Christopher Mungall, Mark Yandell, Lincoln Stein, Richard Durbin, and Michael Ashburner. The sequence ontology: a tool for the unification of genome annotations. *Genome Biology*, 6(5):R44, 2005.

[9] Christine Elsik, Aaron Mackey, Justin Reese, Natalia Milshina, David Roos, and George Weinstock. Creating a honey bee consensus gene set. *Genome Biology*, 8(1):R13, 2007.

[10] National Center for Biotechnology Information. The genbank submissions handbook [internet], 2011.

[11] National Center for Biotechnology Information. Common discrepancy reports, January 2013.

[12] Robert Gentleman, Vincent Carey, Douglas Bates, Ben Bolstad, Marcel Dettling, Sandrine Dudoit, Byron Ellis, Laurent Gautier, Yongchao Ge, Jeff Gentry, Kurt Hornik, Torsten Hothorn, Wolfgang Huber, Stefano Iacus, Rafael Irizarry, Friedrich Leisch, Cheng Li, Martin Maechler, Anthony Rossini, Gunther Sawitzki, Colin

Smith, Gordon Smyth, Luke Tierney, Jean Yang, and Jianhua Zhang. Bioconductor: open software development for computational biology and bioinformatics. *Genome Biology*, 5(10):R80, 2004.

[13] Sante Gnerre, Iain MacCallum, Dariusz Przybylski, Filipe J. Ribeiro, Joshua N. Burton, Bruce J. Walker, Ted Sharpe, Giles Hall, Terrance P. Shea, Sean Sykes, Aaron M. Berlin, Daniel Aird, Maura Costello, Riza Daza, Louise Williams, Robert Nicol, Andreas Gnirke, Chad Nusbaum, Eric S. Lander, and David B. Jaffe. High-quality draft assemblies of mammalian genomes from massively parallel sequence data. *Proceedings of the National Academy of Sciences*, 108(4):1513–1518, 2011.

[14] Brian Haas, Steven Salzberg, Wei Zhu, Mihaela Pertea, Jonathan Allen, Joshua Orvis, Owen White, C Robin Buell, and Jennifer Wortman. Automated eukaryotic gene structure annotation using evidencemodeler and the program to assemble spliced alignments. *Genome Biology*, 9(1):R7, 2008.

[15] Carson Holt and Mark Yandell. Maker2: an annotation pipeline and genome-database management tool for second-generation genome projects. *Bmc Bioinformatics*, 12, 2011.

[16] SE Lewis, SMJ Searle, N Harris, M Gibson, V Iyer, J Richter, C Wiel, L Bayraktaroglu, E Birney, MA Crosby, JS Kaminker, BB Matthews, SE Prochnik, CD Smith, JL Tupy, GM Rubin, S Misra, CJ Mungall, and ME Clamp. Apollo: a sequence annotation editor. *Genome Biology*, 3(12):research0082.1 – 0082.14, 2002.

[17] Michele Magrane and UniProt Consortium. Uniprot knowledgebase: a hub of integrated protein data. *Database*, 2011, 2011.

[18] Barry Moore, Guozhen Fan, and Karen Eilbeck. Soba: sequence ontology bioinformatics analysis. *Nucleic Acids Research*, 38(suppl 2):W161–W164, 2010.

[19] Christopher J. Mungall, Colin Batchelor, and Karen Eilbeck. Evolution of the sequence ontology terms and relationships. *Journal of Biomedical Informatics*, 44(1):87–93, 2011.

[20] Christopher J. Mungall, David B. Emmert, and The FlyBase Consortium. A chado case study: an ontology-based modular schema for representing genome-associated biological information. *Bioinformatics*, 23(13):i337–i346, 2007.

[21] Genome 10K Community of Scientists. Genome 10k: A proposal to obtain whole-genome sequence for 10,000 vertebrate species. *Journal of Heredity*, 100(6):659–674, 2009.

[22] M. Poelchau, C. Childers, G. Moore, V. Tsavatapalli, J. Evans, C. Y. Lee, H. Lin, J. W. Lin, and K. Hackett. The i5k workspace@nal-enabling genomic data access, visualization and curation of arthropod genomes. *Nucleic Acids Research*, 43(D1):D714–D719, 2015.

[23] Martin Reese, Barry Moore, Colin Batchelor, Fidel Salas, Fiona Cunningham, Gabor Marth, Lincoln Stein, Paul Flicek, Mark Yandell, and Karen Eilbeck. A standard variation file format for human genome sequences. *Genome Biology*, 11(8):R88, 2010.

[24] J. T. Robinson, H. Thorvaldsdottir, W. Winckler, M. Guttman, E. S. Lander, G. Getz, and J. P. Mesirov. Integrative genomics viewer. *Nat Biotechnol*, 29(1):24–6, 2011.

[25] K. Rutherford, J. Parkhill, J. Crook, T. Horsnell, P. Rice, M. A. Rajandream, and B. Barrell. Artemis: sequence visualization and annotation. *Bioinformatics*, 16(10):944–5, 2000.

[26] Jared T. Simpson, Kim Wong, Shaun D. Jackman, Jacqueline E. Schein, Steven J.M. Jones, and Inanc Birol. Abyss: A parallel assembler for short read sequence data. *Genome Research*, 19(6):1117–1123, 2009.

[27] Mitchell E. Skinner, Andrew V. Uzilov, Lincoln D. Stein, Christopher J. Mungall, and Ian H. Holmes. Jbrowse: A next-generation genome browser. *Genome Research*, 19(9):1630–1638, 2009.

[28] Jason E. Stajich, David Block, Kris Boulez, Steven E. Brenner, Stephen A. Chervitz, Chris Dagdigian, Georg Fuellen, James G.R. Gilbert, Ian Korf, Hilmar Lapp, Heikki Lehväslaiho, Chad Matsalla, Chris J. Mungall, Brian I. Osborne, Matthew R. Pocock, Peter Schattner, Martin Senger, Lincoln D. Stein, Elia Stupka, Mark D. Wilkinson, and Ewan Birney. The bioperl toolkit: Perl modules for the life sciences. *Genome Research*, 12(10):1611–1618, 2002.

[29] Mario Stanke, Oliver Schoffmann, Burkhard Morgenstern, and Stephan Waack. Gene prediction in eukaryotes with a generalized hidden markov model that uses hints from external sources. *BMC Bioinformatics*, 7(1):62, 2006.

[30] Mario Stanke and Stephan Waack. Gene prediction with a hidden markov model and a new intron submodel. *Bioinformatics*, 19(suppl 2):ii215–ii225, 2003.

[31] Lincoln D. Stein, Christopher Mungall, ShengQiang Shu, Michael Caudy, Marco Mangone, Allen Day, Elizabeth Nickerson, Jason E. Stajich, Todd W. Harris, Adrian Arva, and Suzanna Lewis. The generic genome browser: A building block for a model organism system database. *Genome Research*, 12(10):1599–1610, 2002.

[32] Mark Yandell and Daniel Ence. A beginner's guide to eukaryotic genome annotation. *Nature Reviews Genetics*, 13(5):329–342, 2012.

[33] Evgeni M. Zdobnov and Rolf Apweiler. Interproscan – an integration platform for the signature-recognition methods in interpro. *Bioinformatics*, 17(9):847–848, 2001.
