# Supplementary material for: Genome Annotation Generator: a simple tool for generating and correcting WGS annotation tables for NCBI submission
Source: Gigascience. 2018 Mar 4;7(4):giy018. doi: 10.1093/gigascience/giy018 (PMC5887294; doi:10.1093/gigascience/giy018)
Supplement: GIGA-D-17-00030_Revision_1.pdf [file giy018_giga-d-17-00030_revision_1.pdf]

GigaScience

Genome Annotation Generator: A simple tool for generating and correcting WGS annotation tables for NCBI submission

--Manuscript Draft--

**Manuscript Number:**	GIGA-D-17-00030R1
**Full Title:**	Genome Annotation Generator: A simple tool for generating and correcting WGS annotation tables for NCBI submission
**Article Type:**	Technical Note
**Funding Information:**	Agricultural Research Service — Dr Scott M Geib
**Abstract:**	One of the most overlooked, yet critical components of a whole genome sequencing project is the submission and curation of the data to a genomic repository, most commonly NCBI. While large genome centers or genome groups have developed software tools for post-annotation assembly filtering, annotation, and conversion into NCBI's annotation table format, these tools typically require back-end setup and connection to an SQL database and/or some knowledge of programming (Perl, Python) to implement. With whole genome sequencing becoming commonplace, genome sequencing projects are moving away from the genome centers, and into the ecology or biology lab, where much less resources are present to support the process of genome assembly curation. To fill this gap, we developed software to assess, filter, transfer annotations, and convert a draft genome assembly and annotation set into NCBI annotation table (.tbl) format, facilitating submission to NCBI Genome Assembly database. This software has no dependencies, is compatible across platforms, and utilizes a simple command line to perform a variety of simple and complex post-analysis, pre-NCBI submission WGS project tasks.
**Corresponding Author:**	Scott M Geib<br><br>UNITED STATES
**Corresponding Author Secondary Information:**	
**Corresponding Author's Institution:**	
**Corresponding Author's Secondary Institution:**	
**First Author:**	Scott M Geib
**First Author Secondary Information:**	
**Order of Authors:**	Scott M Geib
	Brian Hall
	Theodore DeRego
	Forest T Bremer
	Kyle Cannoles
	Sheina B Sim
**Order of Authors Secondary Information:**	
**Response to Reviewers:**	Response to Review:<br>Dear Hans Zauner and reviewers. Please find below line-by-line responses to reviewers. We find these reviews very constructive with several good suggestions on improving the manuscript and hopefully also the utility of the tool. If you have any further comments or questions, do not hesitate to contact us.<br>Line-by-line response:<br>Reviewer #1 Comment:<br>This is what it says on the tin, scripts for converting one commonly arising format of files, with some editing and QC and annotation, to another. As the authors say it is

Powered by Editorial Manager® and ProduXion Manager® from Aries Systems Corporation

useful for a particular segment of users, which makes it valuable.
Authors Reply: We appreciate the positive review from this reviewer and did not see any changes that needed to be made based off of their comments.

Reviewer #2 Comments:
The authors describe a command-line-based tool, "Genome Annotation Generator" (GAG) that simplify the task of annotating and formatting new genomes to be submitted to NCBI database. This tool is written in python, it is easy to install and has no external dependencies. Finally, as it uses standard python functions, it is compatible across platforms, provided that python language is previously installed.
The idea of creating a single command-line simplifying a number of tedious tasks is great. However, as more and more options and parameters are added, less simple becomes the tool.
Authors Reply: While there are substantial options for this tool, in its simplest form, only two files (a genome fasta file and annotation gff file) are needed to run the tool, which can be run with no other optional options. Based off of the user's need, that may be all that is required (to generate an NCBI formatted .tbl file). As we developed the tool, users requested various options based off of their needs, and they were integrated into the software. While it does complicate the software a bit (you may run with one or a number of flags), it reduces the need for additional upstream or downstream processing of files (for example to remove short sequences, etc), providing a single command to perform all tasks.

For example, in its current implementation, GAG not only requires a FASTA and a GFF file. In addition, a tab-delimited annotation file (not standard) and a BED file with additional information about regions to be excluded are needed. Although these files are not mandatory, they are usually necessary for fulfilling a proper genome submission procedure. Furthermore, other 18 parameters, related to minimum and maximum genomic feature sizes to be excluded, must be defined by the user with no clear default values provided.
Authors Reply: GAG can be run with only two files, (FASTA and GFF), but if further features are desired, one can utilize them. We chose to include either simple file formats (for example the annotation file is a simple 3 column delimited file, allowing for any text to be added to any feature type, depending on the user's need). To our knowledge, there is no "standard" format for holding this type of data (gene names, products, ontology terms), so an extremely simple to generate format was chosen instead. We also provide scripts (ANNIE package) for generating this delimited file from standard outputs (BLAST, InterProScan, etc). Another extremely common need for a user is to trim scaffold or contig ends. Often during submission to NCBI, they will request removal of ranges due to potential adapter or contaminate sequence, or low quality (lowercase) letters. We utilize another extremely simple file type, a bed file, requiring only 3 columns (feature, start, end). Both the annotation and the bed file can likely be created without knowledge of a programming language using standard bash functions (sed, awk, grep) if the information is contained in some other file format. None of the other features are used, unless selected by the user, so there are no defaults for them (default is not to include that feature in the analysis).

Even when it is true that GAG tool facilitates the task of submitting new genomes to NCBI, it still requires some knowledge of writing command-lines and managing their associated parameters. Including a graphical user interface (GUI) that allows point-and-click events to manage file selections and parameters settings, would be desirable to reach more potential users, not necessarily familiarized with the unix-like console. This GUI would be also helpful to show the user the multiple output files (stats reports, discarded features, session documentation, etc.) that GAG generates and that are very important to check the final quality of the new annotated genome.
Author Reply: We assume if a scientist has assembled and annotated a genome, they would at least have basic skills with command line software, but may not know a programming language. While a GUI may be helpful to some, it may also be a bit clunky. The majority of users of this software we have found are more focused at integrating the command line tool into a genome submission pipeline, rather than requesting a GUI tool. In addition, genome project data is usually very large files, and the ability to run the tool on a compute cluster or ssh into a remote unix machine is probably more desirable than moving the files to a desktop or laptop computer and running the tool through a GUI. We do have future plans to possible integrate this tool

Powered by Editorial Manager® and ProduXion Manager® from Aries Systems Corporation

into a web server, so folks could just browse to a website and covert files, which would ultimately be the best example of what you are requesting, but at the present time, this is outside of the scope of this manuscript.
Finally, a better explanation of example FASTA and GFF files available at "walkthrough/" folder would be desirable. To test the application, this reviewer used the files included in "basic/" subfolder, but other example folders are available (not described).
Author Reply: This is an excellent suggestion, and something we completely overlooked. We have added a section describing the availability of example datasets and provided the walkthrough code and instructions as a supplemental file to this manuscript.

Found some typos:

Page 4. Line 18: "If she..." ---> "If the user..."
Page 4. Line 32: "The to add this level of..." ---> "To add this level of..."
Page 4. Line 60 "teh..." ---> "the..."
Author Reply: We have corrected the typos.

Reviewer #3: Dear Scott, Brian, Theodore, and Sheina,

Thanks for your submission. Your manuscript documents what promises to be a very useful tool for those groups seeking to deposit the fruits of their efforts in genome annotation and curation to NCBI.

Being also a curator myself, I can see the value in the reported work and sincerely hope that you indeed take the steps considered in your conclusions section, so that you may produce an even more versatile tool; specially, when it comes to helping curators in their manual annotation efforts.

I have just a few suggestions for your manuscript, and I hope that you will consider adding these to improve it.
Author Reply: Thank you for your comments

Revisions:

1. In 'Abstract', 'Introduction', and 'Implementation': Of note, I think that the spirit of the narrative may have changed a little as the document progressed; somehow, the 'biologist' with a 'friendly user-interface' you envisioned at the beginning became a 'novice programmer' working on the command line by the end of the manuscript. I am not saying that this is not possible, but rather that it is important to note that, given the manuscript and documentation available on your website, users still need to understand a little more about using the command line than the average field & lab ecologist. Perhaps more care should be given when describing this software as having a 'friendly user-interface' (Page 2, line 55) and 'an intuitive command line program' (page 2, line 53). Although simple, we're still just talking about writing commands in a terminal.
Author Reply: We made our wording consistent throughout to suggest that command line experience is needed, and not overstate simplicity.
1.Page 1, line 49: I would change the text to 'and utilizes a simple command to perform'…
Author Reply: This has been corrected

2. Page 2, Lines 31-33: I am hesitant to encourage the use of blast2go without a warning about using closely related organisms to conduct those searches and propagate functional assignments with them. The result of using blast2go without taking into account the phylogenetic landscape is that many of the annotations propagated may be incorrect, depending in part on the phylogenetic distance to the nearest well-annotated genome. Sequence similarity searches to 'curated databases' by itself, is not enough in this case.
Author Reply: I understand your hesitation with folks generating poor quality functional annotations using automated methods. Our goal here is not to recommend or guide users to a particular program or methodology for generating annotations, rather just

Powered by Editorial Manager® and ProduXion Manager® from Aries Systems Corporation

provide a means for transferring annotations onto GFF/TBL files. We make no clear "encouragement" of blast2go, and don't feel this is a place to guide users on proper usage of functional annotation methods. We simply state that it is a software package that exists (along with many other tools), whose output could be integrated into a genome annotation.

3. Page 2, Lines 31-33: I suggest using the Jones et al. reference (2014) for InterProScan, instead of the ones you use here. See https://www.ncbi.nlm.nih.gov/pmc/articles/PMC3998142/
Author Reply: The reference was updated to Jones et al, 2014

4. Page 3, Line 1: The more appropriate article to reference the efforts of the i5k initiative is the one written by the i5k Consortium, see https://doi.org/10.1093/jhered/est050
Author Reply: The reference was updated

5. In 'Overview' (e.g. Page 3, Lines 18 and 21) and 'Methods' (e.g. Page 4, Line 14): the word 'flag' is used to define both the command used to mark something (e.g. -fis Flag_Introns_Shorter_Than), as well as the action being executed when this command is used (e.g. -ris (Remove_Intron_Shorter_Than). It is a bit redundant and at times confusing. My suggestion is that you use the word 'mark' when you mean that the command you use is going to 'mark' a genomic element with a flag.
Author Reply: We clarified this better in the manuscript, but retain the "flag" terminology to identify items for review, as this has been part of the program for some time and is rather well established.
In 'Overview'

6.1 Page 3, Line 32: Enter ', etc.' after the word 'GBrowse'
Author Reply: "etc." added
6.2 Page 3, Line 32: For reference 16 (Apollo), you should use instead Lee et al 2013. See https://genomebiology.biomedcentral.com/articles/10.1186/gb-2013-14-8-r93 Also, if willing to reference the work of the teams developing JBrowse and others listed, I would also add them to the main text.
Author Reply: The references were updated and sentence added to include JBrowse

7. In 'Methods'

7.1. The GFF3 validator suggested in the documentation available from your GitHub repository points to a tool that is no longer available. Please consider providing other examples, e.g. genometools.org (I found on a quick internet search) seems to work.
Author Reply: The link was updated to genometools.org gff3 validator on the GitHub Page and also recommends genometools gt command line tool as well for gff correction.

7.2. Page 3, Line 43, and in general throughout the document. I have a personal preference to refer to genomic elements as such, or as 'annotations'. I do not use the word 'feature', as I think it carries a meaning more appropriate in the context of software developer and programming. I know it is widely used by many, but I sincerely discourage its use. I would make every effort to discuss 'genomic elements' and 'annotations' instead of 'features'.
Author Reply: While I understand the reviewer's recommendation to use elements and annotations, we chose to retain reference to the feature elements in the documents as "features". This is to maintain consistency with the language used in the guidelines associated with NCBI .tbl format and tbl2asn (@https://www.ncbi.nlm.nih.gov/projects/Sequin/table.html, which we expect users to be using in parallel with this software) and avoid confusion between the two. We did modify language in the manuscript to refer to annotations (when describing annotations of a gene feature) when appropriate (throughout manuscript).
7.3. Page 3, Line 45: Instead of reference [9], please use a more updated version of this work, found at Elsik 2014 (see http://bmcgenomics.biomedcentral.com/articles/10.1186/1471-2164-15-86).
Author Reply: The reference was updated

Powered by Editorial Manager® and ProduXion Manager® from Aries Systems Corporation

7.4. Page 3, Lines 51-56: I think the narrative could be clearer to better illustrate the example. Please consider revising - the text is a bit difficult to follow.
Author Reply: Language was improved

7.5. Page 3. Line 61: How can tbl2asn identify 'low quality sequences' if the user is only providing fasta and gff3 files? Are we to assume somewhere that there are also quality files provided with fasta sequence files?
Author Reply: In this case, this is more of legacy issue, were some older assembly technologies would print low quality assembly as lowercase (if under a defined threshold), or there would be a proportion ambiguous bases (N’s) within a string of sequence. For example, a bedfile could be generated to remove all trailing lowercase bases (e.g. low quality assembly) into scaffold gaps, and the associated GFF3 file coordinates would be updated by GAG.

8. Typos-
8.1 Page 4, Line 5: Typo: please correct - 'infomration'; should be 'information'
Author Reply: We accept your more appropriate spelling of information
8.2 Page 4, Line 19: Typo: should be 'these' criteria.
Author Reply: Corrected
9. Page 4, Line 10-12: important to highlight that although the transcription machinery in eukaryotes more frequently handles introns of at least 50 bp in length, it can also manage with 1bp introns in certain species.
Author Reply: This note was added into the manuscript. It is a bit of a battle with NCBI, with NCBI setting hard cutoffs for minimum sizes of genes, exons, introns, etc (to reduce rate of bad data going into the database) and the exceptions to this that exist in the natural world, that may need some gentle coaxing to get NCBI to accept into their database. Our example is to demonstrate how to get data acceptable to NCBI’s current hard limit, but the caveat that this is the most complete or correct dataset should be considered.

10. Page 4, Lines 28-36: similar to the previous note, if all proteins in the genome should be expected to be at least 50 aa in length, then this is appropriate. Otherwise, a warning should be issued (documented) for curation.
Author Reply: See comment above. GAG does not run with any default cutoff, no cutoff is applied unless supplied by the user. It is up to the user what they see as appropriate to cut (or could flag the feature for manual review).

11. Page 4, Line 40: …"start and stop codons, or if there is reason"… Should this 'or' be an 'and' instead?
Author Reply: This was reworded to be clearer.

12. Page 4, Line 41: Instead of 'calculating' / 'adding' start and stop signals, I think it is more appropriate to say that GAG 'identifies' start and stop sites already in the sequence (as the example in the documentation on your website describes).
Author Reply: This was reworded to be clearer.

13. Page 4, Lines 56-58: Please consider revising fragment for better phrasing. Something along the lines of 'In addition, there may be evidence that certain regions of the assembly are contaminated with microbial, …'
Author Reply: Reworded for clarity and flow

14. I really like that GAG will automatically update coordinates in the .gff3 to reflect any updates to .fasta file!
Author Reply: Thanks, we see this as the central feature of GAG other than writing TBL file

15. Page 4, Line 60: typo: 'teh' should be 'the'.
Author Reply: Corrected
16. Throughout the document, be consistent and decide whether you will use either one or two spaces after periods in the middle of a paragraph.
Author Reply: Document updated to single space between sentences

17. Page 5,

	17.1. Lines 19-35: when you describe the use of 'ontology terms', are you planning to support all available ontologies? Or just GO? The term 'Ontology_term' in the SO does indeed refer to all ontology associations for which a Dbxref exists. Will you also support, for example HPO? Uberon? PATO? etc.<br>Author Reply: Currently we are exclusive to a few db_xref terms. We have put in the feature request pipline to add support for all (or at least most) of the db_xref terms (from here: https://www.ncbi.nlm.nih.gov/genbank/collab/db_xref/)<br><br>17.2. Line 24: Here the reference only cites sequence ontology articles. It should also cite the Gene Ontology (and other supported ontologies). See, https://academic.oup.com/nar/article/45/D1/D331/2605810/Expansion-of-the-Gene-Ontology-knowledgebase-and<br>Author Reply: The reference was updated<br><br>18. Page 8, Line 10: remove text 'Times Cited: 80' from reference [3].<br>Author Reply: The reference was updated<br><br>19. I downloaded and used the software successfully. Also reviewed the code on their repository, which seems stable at this point, with last updates performed back in August of last year. I did not have any problem with executing commands and updating statistics tables.<br>Author Reply: Great!<br><br>20. Page 7, Lines 38-47: The authors have an error in the submitted Table 1. They made a mistake when preparing the table, repeating the explanation for the 'Remove' commands, instead of adding those for the 'Flag' commands. I checked the commands on the software and those are appropriately described there. They just need to update the table accordingly.<br>Author Reply: Table 1 was corrected.
**Additional Information:**	
**Question**	**Response**
Are you submitting this manuscript to a special series or article collection?	No
**Experimental design and statistics**<br><br>Full details of the experimental design and statistical methods used should be given in the Methods section, as detailed in our Minimum Standards Reporting Checklist. Information essential to interpreting the data presented should be made available in the figure legends.<br><br>Have you included all the information requested in your manuscript?	Yes
**Resources**<br><br>A description of all resources used, including antibodies, cell lines, animals and software tools, with enough information to allow them to be uniquely identified, should be included in the Methods section. Authors are strongly encouraged to cite Research Resource Identifiers (RRIDs) for antibodies, model	Yes

Powered by Editorial Manager® and ProduXion Manager® from Aries Systems Corporation

organisms and tools, where possible.<br><br>Have you included the information requested as detailed in our Minimum Standards Reporting Checklist?	
**Availability of data and materials**<br><br>All datasets and code on which the conclusions of the paper rely must be either included in your submission or deposited in publicly available repositories (where available and ethically appropriate), referencing such data using a unique identifier in the references and in the “Availability of Data and Materials” section of your manuscript.<br><br>Have you have met the above requirement as detailed in our Minimum Standards Reporting Checklist?	Yes

Powered by Editorial Manager® and ProduXion Manager® from Aries Systems Corporation

Manuscript

Click here to download Manuscript genome-annotation-generator_Revised.pdf

Genome Annotation Generator: A simple tool for generating and correcting WGS annotation tables for NCBI submission

Scott M. Geib [1*†], Brian Hall [2†], , Theodore Derego [1], Forest T. Bremer,[2], Kyle Cannoles[1,3], and Sheina B. Sim [1]

[1]Tropical Plant Protection Research Unit, USDA-ARS Daniel K. Inouye U.S. Pacific Basin Agricultural Research Center, Hilo, HI, 96720, USA

[2]Plant and Environmental Protection Science, University of Hawaii at Manoa, Honolulu, HI, 96822, USA.

[3]Department of Computer Science and Engineering, University of Hawaii at Hilo, Hilo, HI, 96720, USA.

*Corresponding author †Authors contributed equally

email: SMG: scott.geib@ars.usda.gov BH:bhall7@hawaii.edu TD:t.derego@yahoo.com FTB:forestb@hawaii.edu KC:kylecann@hawaii.edu SBS:sheina.sim@ars.usda.gov

April 12, 2017

Abstract

Background

One of the most overlooked, yet critical components of a whole genome sequencing project is the submission and curation of the data to a genomic repository, most commonly NCBI. While large genome centers or genome groups have developed software tools for post-annotation assembly filtering, annotation, and conversion into NCBI's annotation table format, these tools typically require back-end setup and connection to an SQL database and/or some knowledge of programming (Perl, Python) to implement. With whole genome sequencing becoming commonplace, genome sequencing projects are moving away from the genome centers, and into the ecology or biology lab, where much less resources are present to support the process of genome assembly curation. To fill this gap, we developed software to assess, filter, transfer annotations, and convert a draft genome assembly and annotation set into NCBI annotation table (.tbl) format, facilitating submission to NCBI Genome Assembly database. This software has no dependencies, is compatible across platforms, and utilizes a simple command to perform a variety of simple and complex post-analysis, pre-NCBI submission WGS project tasks.

Findings

The Genome Annotation Generator is a consistent and user-friendly bioinformatics tool that can be used to generate a *.tbl* file that is consistent with the NCBI submission pipeline.

Conclusions

The Genome Annotation Generator achieves the goal of providing a publicly available tool that will facilitate the submission of annotated genome assemblies to NCBI. It is useful for any individual researcher or research group who wishes to submit a genome assembly of their study system to NCBI.

Keywords: Genome curation; annotation; and whole-genome sequencing project

1 Introduction

While ever-improving sequencing technology and assembly software enable the collection of raw sequences for genome assembly and structural annotation, further steps need to be taken to ensure the quality and completeness of a WGS project for submission to the National Center for Biotechnology Information (NCBI) or other data repositories [34]. To submit a genome to the NCBI for curation, it must be converted to the NCBI annotation table format (*.tbl*). With a genome assembly project consisting of thousands of sequences demarcated by hundreds of thousands of structural annotations, this task clearly requires automation. However, there is currently no freely available tool which performs rapid and controlled conversion of a genome assembly and associated structural annotations into a *.tbl* format in addition to allowing for editing, modification, and revision of the content of the project. Moreover, the typical assembly and draft annotation contains some degree of questionable or erroneous data which requires correction or omission. It may also be desirable to add functional annotations to the submission and integrate results from InterProScan, BLAST homology to curated databases, or ontology terms generated by other tools [18, 5, 20].

The traditional approach used to address these problems is to use Linux command line tools or write custom scripts which modify and filter the genome using a scripting language such as Perl or Python [4, 30, 13] or large scale genomic database systems [23]. This method may not be easily or readily reproducible, or it may be entirely beyond the ability of an investigator who has less familiarity with generating custom scripts *de novo*. Even amongst those researchers who use best practices to write clean, well-tested, and reusable scripts to accomplish these tasks, doing requires a large amount of duplicated effort. For this reason, the Genome Annotation Generator (GAG) was written to provide a straightforward and consistent tool for addressing the most common errors in genome assemblies, adding functional annotations from disparate sources, and producing an NCBI submission-ready annotation *.tbl* file. In addition, the software provides a means for integrating existing functional annotations and marking annotations that require manual curation or review. All of these tasks are done through an intuitive command line program requiring only basic unix skills, and has no required dependencies or packages. The program GAG facilitates the submission of whole genome sequencing (WGS) projects to NCBI as well as provide a standardized utility and workflow that fosters consistency

between projects. Due to emerging genome sequencing initiatives such as the 5,000 Insect Genomes Initiative (i5K), the Plant Genome Initiative, and Genome 10K [17, 24], many independent research groups which are not specialized in genome annotation and analysis are generating large genomic datasets and performing genome sequencing projects within their lab. This program can assist in ensuring quality and consistency of data for new genome biologists.

2 Overview

The GAG program is a command line Python program, written in Python 2.7 and requiring no additional outside programs or packages to run. The user directs the program to the genome *.fasta* file and a *.gff3* file containing structure annotations. In addition, a number of options can be used to fix possible errors, flag or remove features (i.e. genomic elements described in the *.gff* structural anntoation file) based on selected criteria, add functional annotations, trim regions of the genome out of the assembly, and, of course, write the genome to NCBI *.tbl* file format. In addition, changes made to the genome annotation, functional annotations added, or flags requesting manual review are also annotated back to the *.gff3* structural annotation file, and the original fasta file is corrected as needed. When the user issues commands to modify the genome, e.g. to remove short introns, the statistics will display two columns, representing the original and modified genomes. This allows for stepwise and documented filtering and review to occur, and interactions between GAG and visual genome review tools (e.g. Artemis, Apollo, GBrowse, JBrowse etc) [33, 27, 19, 29, 26].

3 Methods

As an example, we consider a possible work-flow for a user wishing to prepare a genome for submission to the NCBI Eukaryotic WGS Database. The user has a scaffolded genome assembly produced by one of many whole genome assemblers [2, 14, 28] in *.fasta* file format and a corresponding GFF3 feature file [9, 8] containing structural annotations resulting from an automated annotation pipeline or predictors such as Maker, Evidence Modeler, Jigsaw, or others e.g. [3, 16, 1, 15, 31, 32, 7, 10]. The approach would be to first possibly generate functional annotations of predicted genes if this is desired, using whatever approach the user is interested in, and then using the genome and annotations with GAG. After using GAG to remove or flag features of interest, the user then may then further investigate flagged features in a genome browser by loading the output of GAG, edit, and then perform further filtering in GAG, and iterate through this process until a final draft genome product is generated. Finally GAG writes a NCBI table file, on which *tbl2asn* is run for submission to NCBI. This may identify regions of the genome

that need to be trimmed, due to possible adapter contamination in the genome, or low quality sequence. Any errors generated by *tbl2asn* can then be corrected in GAG, the genome trimmed, until an error free submission is generated.

To use GAG, the user creates a folder containing the genome files (or links to them) and runs *gag.py* from the terminal, with the *.fasta* and *.gff3* files. GAG will write a statistics file, containing infomraiton on the number of each feature type, lengths, and other information that may be useful for the submitter. In our experience, automated genome annotation software frequently produces assemblies containing introns as short as 1 base pair long; if any such features are present, GAG can be run to detect them. It is important to note that while NCBI requires short introns to be removed, cutoffs recommended by NCBI may be more stringent that what you want, as they are set to reduce erroneous data being entered into NCBI. For example, prediction of single base introns might not be errors, and represent true data. It is up to the user to dictate what cutoffs they want to define to remove or flag for manual review. To address these short introns, the user simply applies option *-ris* (Remove_Intron_Shorter_Than) with a value of *10*. GAG will discard any mRNA containing an intron shorter than the minimum of ten. A comparison of the genome content before and after removal is printed to the *.stats* file. If the user instead wishes to only flag features that meet these criteria and not remove them, alternatively the *-fis* (Flag_Introns_Shorter_Than) option could be used, which instead adds a *GAG_FLAG* annotation to the attributes column of the *.gff3* file describing the reason for flagging, allowing manual review of flagged features in a genome browser. GAG will automatically update all parent and child features (gene or CDS entries) to reflect removal of mRNA features. A list of available flag or removal options are listed in Table 1.

Another review for submission might be that all coding regions be a minimum length. For this example we use 150 base pairs in length, which is suggested by NCBI [11, 12]. To add this additional level of filtering, a second option can be used: *-rcs 150*, to Remove_CDS_Shorter_Than 150 bp. When the genome is written to the output folder, GAG will write a file called *genome.removed.gff* containing all the features left out of the final version). It is important to remember that CDS cutoffs at 150 bp will possibly remove some biologically correct amino acids.

GAG supports two straightforward correction, or fix tools. If the user's GFF3 file does not explicitly indicate the presence of start and stop codons, or if there is reason to believe there are errors in ORF prediction in the provided GFF file, GAG can add start and stop feature to the GFF file. The user simply issues the command with the option *–fix_start_stop* and these features will be added to the GFF3 file, and their existence noted in the table file. A second issue that can arise in a draft genome assembly is for a contig or scaffold to have a string of ambiguous bases (N's) at the very beginning or end of the contig. These should be removed from the assembly, and can be using the –

fix_ terminal_ ns option, as they can be mis-interpreted as scaffold gaps. Removing these regions from the genome though, will disrupt the parity between coordinates in the *.fasta* genome file and the *.gff3* annotation file. GAG will automatically update coordinates in the *.gff3* file to reflect any regions removed from the sequence file. During execution of *tbl2asn* or submission to NCBI, it may be identified that regions of the genome may be contaminated with microbial, vector, or sequencing adapter sequence as part of the "contaminate screen" step. A *.bed* formatted file can be supplied with the *-trim* option, containing regions of the assembly to exclude, either ranges within a contig or scaffold, or an entire scaffold. GAG will update both the *.fasta* and *.gff3* files so that coordinate are still synchronized. This is a particularly difficult operation to perform without a specialized tool.

At present, GAG has simple commands to remove or flag introns, exons, coding regions and genes based on minimum or maximum lengths, which will also edit or remove any parent or child feature from the annotation file so as not to create incomplete feature annotations. It can also remove features from a list, which is useful for cases where a genome submission is rejected and a list of invalid mRNAs and genes provided. In addition, all discarded features are retained in a “genome.removed.gff” file and the entire editing session is documented so that the user can retain the filtering criteria used on the particular dataset.

GAG supports two methods to add functional annotations to a genome. First, it can read an annotated GFF3 file containing gene names, protein products, cross-references to databases, and ontology terms following GFF3 qualified nomenclature in the *attribute* column of the GFF3 file [22, 21, 25, 6]. Any annotations present will be automatically carried over to the NCBI feature table file. For users with annotations from another source, GAG can read them from a simple tab-delimited file. The annotations supported by the current version of GAG are *Name* (for genes), *Dbxref*, *Ontology_ term* and *product* (for descriptive mRNA products). These are also written to a new GFF3 file, so GAG can be utilized as a tool to also functionally annotate a GFF3 file. Detailed instructions for running GAG, examples for each of the main functions (e.g. removing features, adding start and stop codons, trimming features, adding annotations) as well as formats and conversion tools for functional annotations are available on the GAG software website webpage: `http://genomeannotation.github.io/GAG/` and as supplemental file 1.

4 Implementation

GAG is written in Python 2.7. It has no dependencies beyond the standard library. The program is modular, abstracting biological concepts such as Sequence, Gene and CDS into classes which may be incorporated into other software tools. In addition, the code is covered by a suite of unit and integration tests, allowing developers to modify or add

to the code base with reduced risk of introducing errors. It should be easily executable by the novice programmer with only basic command-line experience, but also powerful enough to be implemented within robust genomic data processing pipelines.

5 Conclusion

GAG can be easily expanded in the future to support more specific needs of researchers, less common annotation types, and integrate conversion of common functional annotation output formats (e.g. InterProScan, BLAST, Blast2Go) for addition to NCBI annotation table formats. Currently, GAG is an intermediate, but critical tool, between a simple format conversion tool and more sophisticated annotation editors. In future developments of GAG, we plan to allow the integration of multiple lines of evidence supporting gene models to help users discriminate apparently high quality annotations from annotations with little support or possible errors. This could rapidly improve and standardize manual annotation efforts in systems and user groups that are not integrated into genome center annotation pipelines.

6 Declarations

6.1 Competing Interests

The authors declare that they have no competing interests

6.2 Funding

Funding for this project was provided by USDA-ARS and USDA-APHIS Farm Bill Section 10007 projects 3.0251.02 (FY 2014), 3.0256.01 (FY 2015), 3.0392.02 (FY 2016).

6.3 Authors' contributions

SMG conceived software concept. BH, TD, and SMG designed and wrote software. BH, SMG, and SBS wrote manuscript.

6.4 Acknowledgements

We thank S. Gayle, B. Calla, and others for assisting in beta testing of the software and making test datasets available to us. Bioinformatic analysis to develop test datasets for GAG was performed on computing resources at USDA-ARS Pacific Basin Agricultural Research Center (Moana cluster; Hilo, HI) and the Extreme Science and Engineering Discovery Environment (XSEDE), which is supported by National Science Foundation

grant number OCI-1053575XSEDE utilizing allocation TG-MCB140032 to S.M.G. Opinions, findings, conclusions, or recommendations expressed in this publication are those of the authors and do not necessarily reflect the views of the USDA. USDA is an equal opportunity provider and employer

Table 1: **Options for GAG**

Option	Type of function	Description
-*a* <annotation file>	Annotate	Adds functional annotations present in annotation file to *.gff* and *.tbl*
-*t* <*.bed* file>	Trim	Removes regions of genome indicated in *.bed* file from *.fasta* and *.gff3*
-*fix_start_stop* <no value>	Fix	Adds or corrects start and stop codon features to *.gff3*
-*fix_terminal_ns* <no value>	Fix	Removes any trailing ends from contig ends in assembly, updates *.gff3* coordinates
-*rcs* <integer>	Remove	Remove CDS shorter than <integer>
-*rcl* <integer>	Remove	Remove CDS longer than <integer>
-*res* <integer>	Remove	Remove exons shorter than <integer>
-*rel* <integer>	Remove	Remove exons longer than <integer>
-*ris* <integer>	Remove	Remove introns shorter than <integer>
-*ril* <integer>	Remove	Remove introns longer than <integer>
-*rgs* <integer>	Remove	Remove genes shorter than <integer>
-*rgl* <integer>	Remove	Remove genes longer than <integer>
-*fcs* <integer>	Flag	Flag CDS shorter than <integer>
-*fcl* <integer>	Flag	Flag CDS longer than <integer>
-*fes* <integer>	Flag	Flag exons shorter than <integer>
-*fel* <integer>	Flag	Flag exons longer than <integer>
-*fis* <integer>	Flag	Flag introns shorter than <integer>
-*fil* <integer>	Flag	Flag introns longer than <integer>
-*fgs* <integer>	Flag	Flag genes shorter than <integer>
-*fgl* <integer>	Flag	Flag genes longer than <integer>

References

[1] Jonathan E. Allen and Steven L. Salzberg. Jigsaw: integration of multiple sources of evidence for gene prediction. *Bioinformatics*, 21(18):3596–3603, 2005.

[2] J. Butler, I. MacCallum, M. Kleber, I. A. Shlyakhter, M. K. Belmonte, E. S. Lander, C. Nusbaum, and D. B. Jaffe. Allpaths: de novo assembly of whole-genome shotgun microreads. *Genome Res*, 18(5):810–20, 2008.

[3] Brandi L. Cantarel, Ian Korf, Sofia M. C. Robb, Genis Parra, Eric Ross, Barry Moore, Carson Holt, Alejandro Sanchez Alvarado, and Mark Yandell. Maker: An easy-to-use annotation pipeline designed for emerging model organism genomes. *Genome Research*, 18(1):188–196, 2008.

[4] Peter J. A. Cock, Tiago Antao, Jeffrey T. Chang, Brad A. Chapman, Cymon J. Cox, Andrew Dalke, Iddo Friedberg, Thomas Hamelryck, Frank Kauff, Bartek Wilczynski, and Michiel J. L. de Hoon. Biopython: freely available python tools for computational molecular biology and bioinformatics. *Bioinformatics*, 25(11):1422–1423, 2009.

[5] Ana Conesa, Stefan Götz, Juan Miguel García-Gómez, Javier Terol, Manuel Talón, and Montserrat Robles. Blast2go: a universal tool for annotation, visualization and analysis in functional genomics research. *Bioinformatics*, 21(18):3674–3676, 2005.

[6] The Gene Ontology Consortium. Expansion of the gene ontology knowledgebase and resources. *Nucleic Acids Research*, 45(D1):D331–D338, 2017.

[7] Val Curwen, Eduardo Eyras, T. Daniel Andrews, Laura Clarke, Emmanuel Mongin, Steven M.J. Searle, and Michele Clamp. The ensembl automatic gene annotation system. *Genome Research*, 14(5):942–950, 2004.

[8] K. Eilbeck and S. E. Lewis. Sequence ontology annotation guide. *Comp Funct Genomics*, 5(8):642–7, 2004.

[9] Karen Eilbeck, Suzanna Lewis, Christopher Mungall, Mark Yandell, Lincoln Stein, Richard Durbin, and Michael Ashburner. The sequence ontology: a tool for the unification of genome annotations. *Genome Biology*, 6(5):R44, 2005.

[10] Christine G. Elsik, Kim C. Worley, Anna K. Bennett, Martin Beye, Francisco Camara, Christopher P. Childers, Dirk C. de Graaf, Griet Debyser, Jixin Deng, Bart Devreese, Eran Elhaik, Jay D. Evans, Leonard J. Foster, Dan Graur, Roderic Guigo, Katharina Jasmin Hoff, Michael E. Holder, Matthew E. Hudson, Greg J. Hunt, Huaiyang Jiang, Vandita Joshi, Radhika S. Khetani, Peter Kosarev, Christie L.

Kovar, Jian Ma, Ryszard Maleszka, Robin F. A. Moritz, Monica C. Munoz-Torres, Terence D. Murphy, Donna M. Muzny, Irene F. Newsham, Justin T. Reese, Hugh M. Robertson, Gene E. Robinson, Olav Rueppell, Victor Solovyev, Mario Stanke, Eckart Stolle, Jennifer M. Tsuruda, Matthias Van Vaerenbergh, Robert M. Waterhouse, Daniel B. Weaver, Charles W. Whitfield, Yuanqing Wu, Evgeny M. Zdobnov, Lan Zhang, Dianhui Zhu, and Richard A. Gibbs. Finding the missing honey bee genes: lessons learned from a genome upgrade. *BMC Genomics*, 15(1):86, 2014.

[11] National Center for Biotechnology Information. The genbank submissions handbook [internet], 2011.

[12] National Center for Biotechnology Information. Common discrepancy reports, January 2013.

[13] Robert Gentleman, Vincent Carey, Douglas Bates, Ben Bolstad, Marcel Dettling, Sandrine Dudoit, Byron Ellis, Laurent Gautier, Yongchao Ge, Jeff Gentry, Kurt Hornik, Torsten Hothorn, Wolfgang Huber, Stefano Iacus, Rafael Irizarry, Friedrich Leisch, Cheng Li, Martin Maechler, Anthony Rossini, Gunther Sawitzki, Colin Smith, Gordon Smyth, Luke Tierney, Jean Yang, and Jianhua Zhang. Bioconductor: open software development for computational biology and bioinformatics. *Genome Biology*, 5(10):R80, 2004.

[14] Sante Gnerre, Iain MacCallum, Dariusz Przybylski, Filipe J. Ribeiro, Joshua N. Burton, Bruce J. Walker, Ted Sharpe, Giles Hall, Terrance P. Shea, Sean Sykes, Aaron M. Berlin, Daniel Aird, Maura Costello, Riza Daza, Louise Williams, Robert Nicol, Andreas Gnirke, Chad Nusbaum, Eric S. Lander, and David B. Jaffe. High-quality draft assemblies of mammalian genomes from massively parallel sequence data. *Proceedings of the National Academy of Sciences*, 108(4):1513–1518, 2011.

[15] Brian Haas, Steven Salzberg, Wei Zhu, Mihaela Pertea, Jonathan Allen, Joshua Orvis, Owen White, C Robin Buell, and Jennifer Wortman. Automated eukaryotic gene structure annotation using evidencemodeler and the program to assemble spliced alignments. *Genome Biology*, 9(1):R7, 2008.

[16] Carson Holt and Mark Yandell. Maker2: an annotation pipeline and genome-database management tool for second-generation genome projects. *Bmc Bioinformatics*, 12, 2011.

[17] i5K Consortium. The i5k initiative: Advancing arthropod genomics for knowledge, human health, agriculture, and the environment. *Journal of Heredity*, 104(5):595–600, 2013.

[18] Philip Jones, David Binns, Hsin-Yu Chang, Matthew Fraser, Weizhong Li, Craig McAnulla, Hamish McWilliam, John Maslen, Alex Mitchell, Gift Nuka, Sebastien Pesseat, Antony F. Quinn, Amaia Sangrador-Vegas, Maxim Scheremetjew, Siew-Yit Yong, Rodrigo Lopez, and Sarah Hunter. Interproscan 5: genome-scale protein function classification. *Bioinformatics*, 30(9):1236–1240, 2014.

[19] Eduardo Lee, Gregg A. Helt, Justin T. Reese, Monica C. Munoz-Torres, Chris P. Childers, Robert M. Buels, Lincoln Stein, Ian H. Holmes, Christine G. Elsik, and Suzanna E. Lewis. Web apollo: a web-based genomic annotation editing platform. *Genome Biology*, 14(8):R93, 2013.

[20] Michele Magrane and UniProt Consortium. Uniprot knowledgebase: a hub of integrated protein data. *Database*, 2011, 2011.

[21] Barry Moore, Guozhen Fan, and Karen Eilbeck. Soba: sequence ontology bioinformatics analysis. *Nucleic Acids Research*, 38(suppl 2):W161–W164, 2010.

[22] Christopher J. Mungall, Colin Batchelor, and Karen Eilbeck. Evolution of the sequence ontology terms and relationships. *Journal of Biomedical Informatics*, 44(1):87–93, 2011.

[23] Christopher J. Mungall, David B. Emmert, and The FlyBase Consortium. A chado case study: an ontology-based modular schema for representing genome-associated biological information. *Bioinformatics*, 23(13):i337–i346, 2007.

[24] Genome 10K Community of Scientists. Genome 10k: A proposal to obtain whole-genome sequence for 10,000 vertebrate species. *Journal of Heredity*, 100(6):659–674, 2009.

[25] Martin Reese, Barry Moore, Colin Batchelor, Fidel Salas, Fiona Cunningham, Gabor Marth, Lincoln Stein, Paul Flicek, Mark Yandell, and Karen Eilbeck. A standard variation file format for human genome sequences. *Genome Biology*, 11(8):R88, 2010.

[26] J. T. Robinson, H. Thorvaldsdottir, W. Winckler, M. Guttman, E. S. Lander, G. Getz, and J. P. Mesirov. Integrative genomics viewer. *Nat Biotechnol*, 29(1):24–6, 2011.

[27] K. Rutherford, J. Parkhill, J. Crook, T. Horsnell, P. Rice, M. A. Rajandream, and B. Barrell. Artemis: sequence visualization and annotation. *Bioinformatics*, 16(10):944–5, 2000.

[28] Jared T. Simpson, Kim Wong, Shaun D. Jackman, Jacqueline E. Schein, Steven J.M. Jones, and Inanc Birol. Abyss: A parallel assembler for short read sequence data. *Genome Research*, 19(6):1117–1123, 2009.

[29] Mitchell E. Skinner, Andrew V. Uzilov, Lincoln D. Stein, Christopher J. Mungall, and Ian H. Holmes. Jbrowse: A next-generation genome browser. *Genome Research*, 19(9):1630–1638, 2009.

[30] Jason E. Stajich, David Block, Kris Boulez, Steven E. Brenner, Stephen A. Chervitz, Chris Dagdigian, Georg Fuellen, James G.R. Gilbert, Ian Korf, Hilmar Lapp, Heikki Lehväslaiho, Chad Matsalla, Chris J. Mungall, Brian I. Osborne, Matthew R. Pocock, Peter Schattner, Martin Senger, Lincoln D. Stein, Elia Stupka, Mark D. Wilkinson, and Ewan Birney. The bioperl toolkit: Perl modules for the life sciences. *Genome Research*, 12(10):1611–1618, 2002.

[31] Mario Stanke, Oliver Schoffmann, Burkhard Morgenstern, and Stephan Waack. Gene prediction in eukaryotes with a generalized hidden markov model that uses hints from external sources. *BMC Bioinformatics*, 7(1):62, 2006.

[32] Mario Stanke and Stephan Waack. Gene prediction with a hidden markov model and a new intron submodel. *Bioinformatics*, 19(suppl 2):ii215–ii225, 2003.

[33] Lincoln D. Stein, Christopher Mungall, ShengQiang Shu, Michael Caudy, Marco Mangone, Allen Day, Elizabeth Nickerson, Jason E. Stajich, Todd W. Harris, Adrian Arva, and Suzanna Lewis. The generic genome browser: A building block for a model organism system database. *Genome Research*, 12(10):1599–1610, 2002.

[34] Mark Yandell and Daniel Ence. A beginner's guide to eukaryotic genome annotation. *Nature Reviews Genetics*, 13(5):329–342, 2012.
