# Supplementary material for: Genome Annotation Generator: a simple tool for generating and correcting WGS annotation tables for NCBI submission
Source: Gigascience. 2018 Mar 4;7(4):giy018. doi: 10.1093/gigascience/giy018 (PMC5887294; doi:10.1093/gigascience/giy018)
Supplement: Response_to_Reviewer_Comments_Original_Submission.pdf [file giy018_response_to_reviewer_comments_original_submission.pdf]

Author's Response To Reviewer Comments

Response to Review:
Dear Hans Zauner and reviewers. Please find below line-by-line responses to reviewers. We find these reviews very constructive with several good suggestions on improving the manuscript and hopefully also the utility of the tool. If you have any further comments or questions, do not hesitate to contact us.
Line-by-line response:
Reviewer #1 Comment:
This is what it says on the tin, scripts for converting one commonly arising format of files, with some editing and QC and annotation, to another. As the authors say it is useful for a particular segment of users, which makes it valuable.
Authors Reply: We appreciate the positive review from this reviewer and did not see any changes that needed to be made based off of their comments.

Reviewer #2 Comments:
The authors describe a command-line-based tool, "Genome Annotation Generator" (GAG) that simplify the task of annotating and formatting new genomes to be submitted to NCBI database. This tool is written in python, it is easy to install and has no external dependencies. Finally, as it uses standard python functions, it is compatible across platforms, provided that python language is previously installed.
The idea of creating a single command-line simplifying a number of tedious tasks is great. However, as more and more options and parameters are added, less simple becomes the tool.
Authors Reply: While there are substantial options for this tool, in its simplest form, only two files (a genome fasta file and annotation gff file) are needed to run the tool, which can be run with no other optional options. Based off of the user's need, that may be all that is required (to generate an NCBI formatted .tbl file). As we developed the tool, users requested various options based off of their needs, and they were integrated into the software. While it does complicate the software a bit (you may run with one or a number of flags), it reduces the need for additional upstream or downstream processing of files (for example to remove short sequences, etc), providing a single command to perform all tasks.

For example, in its current implementation, GAG not only requires a FASTA and a GFF file. In addition, a tab-delimited annotation file (not standard) and a BED file with additional information about regions to be excluded are needed. Although these files are not mandatory, they are usually necessary for fulfilling a proper genome submission procedure. Furthermore, other 18 parameters, related to minimum and maximum genomic feature sizes to be excluded, must be defined by the user with no clear default values provided.
Authors Reply: GAG can be run with only two files, (FASTA and GFF), but if further features are desired, one can utilize them. We chose to include either simple file formats (for example the annotation file is a simple 3 column delimited file, allowing for any text to be added to any feature type, depending on the user's need). To our knowledge, there is no "standard" format for holding this type of data (gene names, products, ontology terms), so an extremely simple to generate format was chosen instead. We also provide scripts (ANNIE package) for generating this delimited file from standard outputs (BLAST, InterProScan, etc). Another extremely common need for a user is to trim scaffold or contig ends. Often during submission to NCBI,

they will request removal of ranges due to potential adapter or contaminate sequence, or low quality (lowercase) letters. We utilize another extremely simple file type, a bed file, requiring only 3 columns (feature, start, end). Both the annotation and the bed file can likely be created without knowledge of a programming language using standard bash functions (sed, awk, grep) if the information is contained in some other file format. None of the other features are used, unless selected by the user, so there are no defaults for them (default is not to include that feature in the analysis).

Even when it is true that GAG tool facilitates the task of submitting new genomes to NCBI, it still requires some knowledge of writing command-lines and managing their associated parameters. Including a graphical user interface (GUI) that allows point-and-click events to manage file selections and parameters settings, would be desirable to reach more potential users, not necessarily familiarized with the unix-like console. This GUI would be also helpful to show the user the multiple output files (stats reports, discarded features, session documentation, etc.) that GAG generates and that are very important to check the final quality of the new annotated genome.
Author Reply: We assume if a scientist has assembled and annotated a genome, they would at least have basic skills with command line software, but may not know a programming language. While a GUI may be helpful to some, it may also be a bit clunky. The majority of users of this software we have found are more focused at integrating the command line tool into a genome submission pipeline, rather than requesting a GUI tool. In addition, genome project data is usually very large files, and the ability to run the tool on a compute cluster or ssh into a remote unix machine is probably more desirable than moving the files to a desktop or laptop computer and running the tool through a GUI. We do have future plans to possible integrate this tool into a web server, so folks could just browse to a website and covert files, which would ultimately be the best example of what you are requesting, but at the present time, this is outside of the scope of this manuscript.
Finally, a better explanation of example FASTA and GFF files available at "walkthrough/" folder would be desirable. To test the application, this reviewer used the files included in "basic/" subfolder, but other example folders are available (not described).
Author Reply: This is an excellent suggestion, and something we completely overlooked. We have added a section describing the availability of example datasets and provided the walkthrough code and instructions as a supplemental file to this manuscript.

Found some typos:

Page 4. Line 18: "If she..." ---> "If the user..."
Page 4. Line 32: "The to add this level of..." ---> "To add this level of..."
Page 4. Line 60 "teh..." ---> "the..."
Author Reply: We have corrected the typos.

Reviewer #3: Dear Scott, Brian, Theodore, and Sheina,

Thanks for your submission. Your manuscript documents what promises to be a very useful tool for those groups seeking to deposit the fruits of their efforts in genome annotation and curation to

NCBI.

Being also a curator myself, I can see the value in the reported work and sincerely hope that you indeed take the steps considered in your conclusions section, so that you may produce an even more versatile tool; specially, when it comes to helping curators in their manual annotation efforts.

I have just a few suggestions for your manuscript, and I hope that you will consider adding these to improve it.
Author Reply: Thank you for your comments

Revisions:

1. In 'Abstract', 'Introduction', and 'Implementation': Of note, I think that the spirit of the narrative may have changed a little as the document progressed; somehow, the 'biologist' with a 'friendly user-interface' you envisioned at the beginning became a 'novice programmer' working on the command line by the end of the manuscript. I am not saying that this is not possible, but rather that it is important to note that, given the manuscript and documentation available on your website, users still need to understand a little more about using the command line than the average field & lab ecologist. Perhaps more care should be given when describing this software as having a 'friendly user-interface' (Page 2, line 55) and 'an intuitive command line program' (page 2, line 53). Although simple, we're still just talking about writing commands in a terminal.
Author Reply: We made our wording consistent throughout to suggest that command line experience is needed, and not overstate simplicity.
1. Page 1, line 49: I would change the text to 'and utilizes a simple command to perform'…
Author Reply: This has been corrected

2. Page 2, Lines 31-33: I am hesitant to encourage the use of blast2go without a warning about using closely related organisms to conduct those searches and propagate functional assignments with them. The result of using blast2go without taking into account the phylogenetic landscape is that many of the annotations propagated may be incorrect, depending in part on the phylogenetic distance to the nearest well-annotated genome. Sequence similarity searches to 'curated databases' by itself, is not enough in this case.
Author Reply: I understand your hesitation with folks generating poor quality functional annotations using automated methods. Our goal here is not to recommend or guide users to a particular program or methodology for generating annotations, rather just provide a means for transferring annotations onto GFF/TBL files. We make no clear “encouragement” of blast2go, and don’t feel this is a place to guide users on proper usage of functional annotation methods. We simply state that it is a software package that exists (along with many other tools), whose output could be integrated into a genome annotation.

3. Page 2, Lines 31-33: I suggest using the Jones et al. reference (2014) for InterProScan, instead of the ones you use here. See https://www.ncbi.nlm.nih.gov/pmc/articles/PMC3998142/
Author Reply: The reference was updated to Jones et al, 2014

4. Page 3, Line 1: The more appropriate article to reference the efforts of the i5k initiative is the

one written by the i5k Consortium, see https://doi.org/10.1093/jhered/est050
Author Reply: The reference was updated

5. In 'Overview' (e.g. Page 3, Lines 18 and 21) and 'Methods' (e.g. Page 4, Line 14): the word 'flag' is used to define both the command used to mark something (e.g. -fis Flag_Introns_Shorter_Than), as well as the action being executed when this command is used (e.g. -ris (Remove_Intron_Shorter_Than). It is a bit redundant and at times confusing. My suggestion is that you use the word 'mark' when you mean that the command you use is going to 'mark' a genomic element with a flag.
Author Reply: We clarified this better in the manuscript, but retain the “flag” terminology to identify items for review, as this has been part of the program for some time and is rather well established.
In 'Overview'

6.1 Page 3, Line 32: Enter ', etc.' after the word 'GBrowse'
Author Reply: “etc.” added
6.2 Page 3, Line 32: For reference 16 (Apollo), you should use instead Lee et al 2013. See https://genomebiology.biomedcentral.com/articles/10.1186/gb-2013-14-8-r93 Also, if willing to reference the work of the teams developing JBrowse and others listed, I would also add them to the main text.
Author Reply: The references were updated and sentence added to include JBrowse

7. In 'Methods'

7.1. The GFF3 validator suggested in the documentation available from your GitHub repository points to a tool that is no longer available. Please consider providing other examples, e.g. genometools.org (I found on a quick internet search) seems to work.
Author Reply: The link was updated to genometools.org gff3 validator on the GitHub Page and also recommends genometools gt command line tool as well for gff correction.

7.2. Page 3, Line 43, and in general throughout the document. I have a personal preference to refer to genomic elements as such, or as 'annotations'. I do not use the word 'feature', as I think it carries a meaning more appropriate in the context of software developer and programming. I know it is widely used by many, but I sincerely discourage its use. I would make every effort to discuss 'genomic elements' and 'annotations' instead of 'features'.
Author Reply: While I understand the reviewer’s recommendation to use elements and annotations, we chose to retain reference to the feature elements in the documents as “features”. This is to maintain consistency with the language used in the guidelines associated with NCBI .tbl format and tbl2asn (@https://www.ncbi.nlm.nih.gov/projects/Sequin/table.html, which we expect users to be using in parallel with this software) and avoid confusion between the two. We did modify language in the manuscript to refer to annotations (when describing annotations of a gene feature) when appropriate (throughout manuscript).
7.3. Page 3, Line 45: Instead of reference [9], please use a more updated version of this work, found at Elsik 2014 (see http://bmcgenomics.biomedcentral.com/articles/10.1186/1471-2164-15-86).

Author Reply: The reference was updated

7.4. Page 3, Lines 51-56: I think the narrative could be clearer to better illustrate the example. Please consider revising - the text is a bit difficult to follow.
Author Reply: Language was improved

7.5. Page 3. Line 61: How can tbl2asn identify 'low quality sequences' if the user is only providing fasta and gff3 files? Are we to assume somewhere that there are also quality files provided with fasta sequence files?
Author Reply: In this case, this is more of legacy issue, were some older assembly technologies would print low quality assembly as lowercase (if under a defined threshold), or there would be a proportion ambiguous bases (N's) within a string of sequence. For example, a bedfile could be generated to remove all trailing lowercase bases (e.g. low quality assembly) into scaffold gaps, and the associated GFF3 file coordinates would be updated by GAG.

8. Typos-
8.1 Page 4, Line 5: Typo: please correct - 'infomration'; should be 'information'
Author Reply: We accept your more appropriate spelling of information
8.2 Page 4, Line 19: Typo: should be 'these' criteria.
Author Reply: Corrected
9. Page 4, Line 10-12: important to highlight that although the transcription machinery in eukaryotes more frequently handles introns of at least 50 bp in length, it can also manage with 1bp introns in certain species.
Author Reply: This note was added into the manuscript. It is a bit of a battle with NCBI, with NCBI setting hard cutoffs for minimum sizes of genes, exons, introns, etc (to reduce rate of bad data going into the database) and the exceptions to this that exist in the natural world, that may need some gentle coaxing to get NCBI to accept into their database. Our example is to demonstrate how to get data acceptable to NCBI's current hard limit, but the caveat that this is the most complete or correct dataset should be considered.

10. Page 4, Lines 28-36: similar to the previous note, if all proteins in the genome should be expected to be at least 50 aa in length, then this is appropriate. Otherwise, a warning should be issued (documented) for curation.
Author Reply: See comment above. GAG does not run with any default cutoff, no cutoff is applied unless supplied by the user. It is up to the user what they see as appropriate to cut (or could flag the feature for manual review).

11. Page 4, Line 40: …"start and stop codons, or if there is reason"… Should this 'or' be an 'and' instead?
Author Reply: This was reworded to be clearer.

12. Page 4, Line 41: Instead of 'calculating' / 'adding' start and stop signals, I think it is more appropriate to say that GAG 'identifies' start and stop sites already in the sequence (as the example in the documentation on your website describes).
Author Reply: This was reworded to be clearer.

13. Page 4, Lines 56-58: Please consider revising fragment for better phrasing. Something along the lines of 'In addition, there may be evidence that certain regions of the assembly are contaminated with microbial, …'
Author Reply: Reworded for clarity and flow

14. I really like that GAG will automatically update coordinates in the .gff3 to reflect any updates to .fasta file!
Author Reply: Thanks, we see this as the central feature of GAG other than writing TBL file

15. Page 4, Line 60: typo: 'teh' should be 'the'.
Author Reply: Corrected
16. Throughout the document, be consistent and decide whether you will use either one or two spaces after periods in the middle of a paragraph.
Author Reply: Document updated to single space between sentences

17. Page 5,

17.1. Lines 19-35: when you describe the use of 'ontology terms', are you planning to support all available ontologies? Or just GO? The term 'Ontology_term' in the SO does indeed refer to all ontology associations for which a Dbxref exists. Will you also support, for example HPO? Uberon? PATO? etc.
Author Reply: Currently we are exclusive to a few db_xref terms. We have put in the feature request pipline to add support for all (or at least most) of the db_xref terms (from here: https://www.ncbi.nlm.nih.gov/genbank/collab/db_xref/)

17.2. Line 24: Here the reference only cites sequence ontology articles. It should also cite the Gene Ontology (and other supported ontologies). See, https://academic.oup.com/nar/article/45/D1/D331/2605810/Expansion-of-the-Gene-Ontology-knowledgebase-and
Author Reply: The reference was updated

18. Page 8, Line 10: remove text 'Times Cited: 80' from reference [3].
Author Reply: The reference was updated

19. I downloaded and used the software successfully. Also reviewed the code on their repository, which seems stable at this point, with last updates performed back in August of last year. I did not have any problem with executing commands and updating statistics tables.
Author Reply: Great!

20. Page 7, Lines 38-47: The authors have an error in the submitted Table 1. They made a mistake when preparing the table, repeating the explanation for the 'Remove' commands, instead of adding those for the 'Flag' commands. I checked the commands on the software and those are appropriately described there. They just need to update the table accordingly.
Author Reply: Table 1 was corrected.
