# Supplementary material for: Genome Annotation Generator: a simple tool for generating and correcting WGS annotation tables for NCBI submission
Source: Gigascience. 2018 Mar 4;7(4):giy018. doi: 10.1093/gigascience/giy018 (PMC5887294; doi:10.1093/gigascience/giy018)
Supplement: Response_to_Reviewer_Comments_Revision_1.pdf [file giy018_response_to_reviewer_comments_revision_1.pdf]

Author's Response To Reviewer Comments

Hi, I have modified the manuscript to reference a GigaDB entry with a stable release for software access rather than just referencing a github page
