# Supplementary material for: Genome Annotation Generator: a simple tool for generating and correcting WGS annotation tables for NCBI submission
Source: Gigascience. 2018 Mar 4;7(4):giy018. doi: 10.1093/gigascience/giy018 (PMC5887294; doi:10.1093/gigascience/giy018)
Supplement: Reviewer_2_Report_(Original_Submission) -- Juan Oliveros — 08 Mar 2017 Reviewed [file giy018_reviewer_2_report_(original_submission).pdf]

Reviewer Report

Title: Genome Annotation Generator: A simple tool for generating and correcting WGS annotation tables for NCBI submission

Version: Original Submission **Date:** 08 Mar 2017

Reviewer name: Juan Oliveros

Reviewer Comments to Author:

The authors describe a command-line-based tool, "Genome Annotation Generator" (GAG) that simplify the task of annotating and formatting new genomes to be submitted to NCBI database. This tool is written in python, it is easy to install and has no external dependencies. Finally, as it uses standard python functions, it is compatible across platforms, provided that python language is previously installed.

The idea of creating a single command-line simplifying a number of tedious tasks is great. However, as more and more options and parameters are added, less simple becomes the tool.

For example, in its current implementation, GAG not only requires a FASTA and a GFF file. In addition, a tab-delimited annotation file (not standard) and a BED file with additional information about regions to be excluded are needed. Although these files are not mandatory, they are usually necessary for fulfilling a proper genome submission procedure. Furthermore, other 18 parameters, related to minimum and maximum genomic feature sizes to be excluded, must be defined by the user with no clear default values provided.

Even when it is true that GAG tool facilitates the task of submitting new genomes to NCBI, it still requires some knowledge of writing command-lines and managing their associated parameters. Including a graphical user interface (GUI) that allows point-and-click events to manage file selections and parameters settings, would be desirable to reach more potential users, not necessarily familiarized with the unix-like console. This GUI would be also helpful to show the user the multiple output files (stats reports, discarded features, session documentation, etc.) that GAG generates and that are very important to check the final quality of the new annotated genome.

Finally, a better explanation of example FASTA and GFF files available at "walkthrough/" folder would be desirable. To test the application, this reviewer used the files included in "basic/" subfolder, but other example folders are available (not described).

Found some typos:

Page 4. Line 18: "If she..." ---> "If the user..."

Page 4. Line 32: "The to add this level of..." ---> "To add this level of..."
Page 4. Line 60 "teh..." ---> "the..."

Level of Interest

Please indicate how interesting you found the manuscript: An article whose findings are important to those with closely related research interests

Quality of Written English

Please indicate the quality of language in the manuscript: Acceptable

Declaration of Competing Interests

Please complete a declaration of competing interests, considering the following questions:

- Have you in the past five years received reimbursements, fees, funding, or salary from an organisation that may in any way gain or lose financially from the publication of this manuscript, either now or in the future?
- Do you hold any stocks or shares in an organisation that may in any way gain or lose financially from the publication of this manuscript, either now or in the future?
- Do you hold or are you currently applying for any patents relating to the content of the manuscript?
- Have you received reimbursements, fees, funding, or salary from an organization that holds or has applied for patents relating to the content of the manuscript?
- Do you have any other financial competing interests?
- Do you have any non-financial competing interests in relation to this paper?

If you can answer no to all of the above, write 'I declare that I have no competing interests' below. If your reply is yes to any, please give details below.

I declare that I have no competing interests

I agree to the open peer review policy of the journal. I understand that my name will be included on my report to the authors and, if the manuscript is accepted for publication, my named report including any attachments I upload will be posted on the website along with the authors' responses. I agree for my report to be made available under an Open Access Creative Commons CC-BY license (http://creativecommons.org/licenses/by/4.0/). I understand that any comments which I do not wish to

be included in my named report can be included as confidential comments to the editors, which will not be published.

I agree to the open peer review policy of the journal

To further support our reviewers, we have joined with Publons, where you can gain additional credit to further highlight your hard work (see: https://publons.com/journal/530/gigascience). On publication of this paper, your review will be automatically added to Publons, you can then choose whether or not to claim your Publons credit. I understand this statement.

Yes
