# Supplementary material for: Genome Annotation Generator: a simple tool for generating and correcting WGS annotation tables for NCBI submission
Source: Gigascience. 2018 Mar 4;7(4):giy018. doi: 10.1093/gigascience/giy018 (PMC5887294; doi:10.1093/gigascience/giy018)
Supplement: Reviewer_3_Report_(Original_Submission) -- Monica Munoz-Torres — 15 Mar 2017 Reviewed [file giy018_reviewer_3_report_(original_submission).pdf]

Reviewer Report

Title: Genome Annotation Generator: A simple tool for generating and correcting WGS annotation tables for NCBI submission

Version: Original Submission **Date:** 15 Mar 2017

Reviewer name: Monica Munoz-Torres

Reviewer Comments to Author:

Dear Scott, Brian, Theodore, and Sheina,

Thanks for your submission. Your manuscript documents what promises to be a very useful tool for those groups seeking to deposit the fruits of their efforts in genome annotation and curation to NCBI.

Being also a curator myself, I can see the value in the reported work and sincerely hope that you indeed take the steps considered in your conclusions section, so that you may produce an even more versatile tool; specially, when it comes to helping curators in their manual annotation efforts.

I have just a few suggestions for your manuscript, and I hope that you will consider adding these to improve it.

Revisions:

1. In 'Abstract', 'Introduction', and 'Implementation': Of note, I think that the spirit of the narrative may have changed a little as the document progressed; somehow, the 'biologist' with a 'friendly user-interface' you envisioned at the beginning became a 'novice programmer' working on the command line by the end of the manuscript. I am not saying that this is not possible, but rather that it is important to note that, given the manuscript and documentation available on your website, users still need to understand a little more about using the command line than the average field & lab ecologist. Perhaps more care should be given when describing this software as having a 'friendly user-interface' (Page 2, line 55) and 'an intuitive command line program' (page 2, line 53). Although simple, we're still just talking about writing commands in a terminal.

1.1. Page 1, line 49: I would change the text to 'and utilizes a simple command to perform'...

2. Page 2, Lines 31-33: I am hesitant to encourage the use of blast2go without a warning about using closely related organisms to conduct those searches and propagate functional assignments with them. The result of using blast2go without taking into account the phylogenetic landscape is that many of the annotations propagated may be incorrect, depending in part on the phylogenetic distance to the nearest well-annotated genome. Sequence similarity searches to 'curated databases' by itself, is not enough in this case.

3. Page 2, Lines 31-33: I suggest using the Jones et al. reference (2014) for InterProScan, instead of the ones you use here. See https://www.ncbi.nlm.nih.gov/pmc/articles/PMC3998142/

4. Page 3, Line 1: The more appropriate article to reference the efforts of the i5k initiative is the one written by the i5k Consortium, see https://doi.org/10.1093/jhered/est050

5. In 'Overview' (e.g. Page 3, Lines 18 and 21) and 'Methods' (e.g. Page 4, Line 14): the word 'flag' is used to define both the command used to mark something (e.g. -fis Flag_Introns_Shorter_Than), as well as the action being executed when this command is used (e.g. -ris (Remove_Intron_Shorter_Than). It is a bit redundant and at times confusing. My suggestion is that you use the word 'mark' when you mean that the command you use is going to 'mark' a genomic element with a flag.

6. In 'Overview'

6.1 Page 3, Line 32: Enter ', etc.' after the word 'GBrowse'

6.2 Page 3, Line 32: For reference 16 (Apollo), you should use instead Lee et al 2013. See https://genomebiology.biomedcentral.com/articles/10.1186/gb-2013-14-8-r93 Also, if willing to reference the work of the teams developing JBrowse and others listed, I would also add them to the main text.

7. In 'Methods'

7.1. The GFF3 validator suggested in the documentation available from your GitHub repository points to a tool that is no longer available. Please consider providing other examples, e.g. genometools.org (I found on a quick internet search) seems to work.

7.2. Page 3, Line 43, and in general throughout the document. I have a personal preference to refer to genomic elements as such, or as 'annotations'. I do not use the word 'feature', as I think it carries a meaning more appropriate in the context of software developer and programming. I know it is widely used by many, but I sincerely discourage its use. I would make every effort to discuss 'genomic elements' and 'annotations' instead of 'features'.

7.3. Page 3, Line 45: Instead of reference [9], please use a more updated version of this work, found at Elsik 2014 (see http://bmcgenomics.biomedcentral.com/articles/10.1186/1471-2164-15-86).

7.4. Page 3, Lines 51-56: I think the narrative could be clearer to better illustrate the example. Please consider revising - the text is a bit difficult to follow.

7.5. Page 3. Line 61: How can tbl2asn identify 'low quality sequences' if the user is only providing fasta and gff3 files? Are we to assume somewhere that there are also quality files provided with fasta

sequence files?

8. Typos-
8.1 Page 4, Line 5: Typo: please correct - 'infomration'; should be 'information'
8.2 Page 4, Line 19: Typo: should be 'these' criteria.

9. Page 4, Line 10-12: important to highlight that although the transcription machinery in eukaryotes more frequently handles introns of at least 50 bp in length, it can also manage with 1bp introns in certain species.

10. Page 4, Lines 28-36: similar to the previous note, if all proteins in the genome should be expected to be at least 50 aa in length, then this is appropriate. Otherwise, a warning should be issued (documented) for curation.

11. Page 4, Line 40: ..."start and stop codons, or if there is reason"... Should this 'or' be an 'and' instead?

12. Page 4, Line 41: Instead of 'calculating' / 'adding' start and stop signals, I think it is more appropriate to say that GAG 'identifies' start and stop sites already in the sequence (as the example in the documentation on your website describes).

13. Page 4, Lines 56-58: Please consider revising fragment for better phrasing. Something along the lines of 'In addition, there may be evidence that certain regions of the assembly are contaminated with microbial, ...'

14. I really like that GAG will automatically update coordinates in the .gff3 to reflect any updates to .fasta file!

15. Page 4, Line 60: typo: 'teh' should be 'the'.

16. Throughout the document, be consistent and decide whether you will use either one or two spaces after periods in the middle of a paragraph.

17. Page 5,

17.1. Lines 19-35: when you describe the use of 'ontology terms', are you planning to support all available ontologies? Or just GO? The term 'Ontology_term' in the SO does indeed refer to all ontology associations for which a Dbxref exists. Will you also support, for example HPO? Uberon? PATO? etc.

17.2. Line 24: Here the reference only cites sequence ontology articles. It should also cite the Gene Ontology (and other supported ontologies). See, https://academic.oup.com/nar/article/45/D1/D331/2605810/Expansion-of-the-Gene-Ontology-knowledgebase-and

18. Page 8, Line 10: remove text 'Times Cited: 80' from reference [3].

19. I downloaded and used the software successfully. Also reviewed the code on their repository, which seems stable at this point, with last updates performed back in August of last year. I did not have any problem with executing commands and updating statistics tables.

20. Page 7, Lines 38-47: The authors have an error in the submitted Table 1. They made a mistake when preparing the table, repeating the explanation for the 'Remove' commands, instead of adding those for the 'Flag' commands. I checked the commands on the software and those are appropriately described there. They just need to update the table accordingly.

Level of Interest

Please indicate how interesting you found the manuscript: An article whose findings are important to those with closely related research interests

Quality of Written English

Please indicate the quality of language in the manuscript: Acceptable

Declaration of Competing Interests

Please complete a declaration of competing interests, considering the following questions:

- Have you in the past five years received reimbursements, fees, funding, or salary from an organisation that may in any way gain or lose financially from the publication of this manuscript, either now or in the future?
- Do you hold any stocks or shares in an organisation that may in any way gain or lose financially from the publication of this manuscript, either now or in the future?
- Do you hold or are you currently applying for any patents relating to the content of the manuscript?
- Have you received reimbursements, fees, funding, or salary from an organization that holds or has applied for patents relating to the content of the manuscript?
- Do you have any other financial competing interests?
- Do you have any non-financial competing interests in relation to this paper?

If you can answer no to all of the above, write 'I declare that I have no competing interests' below. If your reply is yes to any, please give details below.

I declare that I have no competing interests.

I agree to the open peer review policy of the journal. I understand that my name will be included on my report to the authors and, if the manuscript is accepted for publication, my named report including any attachments I upload will be posted on the website along with the authors' responses. I agree for my report to be made available under an Open Access Creative Commons CC-BY license (http://creativecommons.org/licenses/by/4.0/). I understand that any comments which I do not wish to be included in my named report can be included as confidential comments to the editors, which will not be published.

I agree to the open peer review policy of the journal

To further support our reviewers, we have joined with Publons, where you can gain additional credit to further highlight your hard work (see: https://publons.com/journal/530/gigascience). On publication of this paper, your review will be automatically added to Publons, you can then choose whether or not to claim your Publons credit. I understand this statement.

Yes
